# Data-independent acquisition-based blood proteomics unveils predictive biomarkers for neonatal necrotizing enterocolitis

**DOI:** 10.1007/s00216-024-05637-7

**Published:** 2024-11-20

**Authors:** Feng Chen, Kezhe Tan, Zhibao Lv, Faling Chen, Weijue Xu, Xiaohui Gong, Li Lu, Hailiang Sun, Qinqin Fu, Wenjun Zhuang

**Affiliations:** 1 Department of General Surgery, Shanghai Children’s Hospital, School of Medicine, Shanghai Jiao Tong University, Shanghai, China; 2 Department of Neonatology, Shanghai Children’s Hospital, School of Medicine, Shanghai Jiao Tong University, Shanghai, China; 3 Department of General Surgery, Affiliated Changzhou Children’s Hospital of Nantong University, Jiangsu, China; 4 Department of Neonatology, Huzhou Maternity & Child Health Care Hospital, Zhejiang, China

**Keywords:** DIA mass spectrometry, Proteomics, NEC, DEPs, Clinical and biological relevance

## Abstract

Necrotizing enterocolitis (NEC) is a life-threatening condition affecting preterm infants, sometimes necessitating surgical treatment. This study aimed to analyze differentially expressed proteins (DEPs) and access their biological and clinical significance in the plasma of neonates with NEC. Peripheral blood samples were collected from NEC infants at various time points, and plasma was separated. Data-independent acquisition (DIA) technology was utilized to identify DEPs among NEC patients at different stages. Bioinformatic analyses, including Gene Ontology and Kyoto Encyclopedia of Genes and Genomes, and protein-to-protein interaction analyses were performed on the DEPs. External datasets, along with receiver operating characteristic curves and gene set enrichment analysis, were used to clinically and biologically validate the findings. DEPs between the NEC and pre-NEC groups indicated reduced protein, heme, nitrogen, and purine nucleotide biosynthesis during NEC formation. In addition, enriched DEPs among the NEC groups at different time points suggested reconstructed extracellular matrix, aberrant B-lymphocyte immune responses, and decreased glycosaminoglycan levels during NEC progression. These findings were both clinically and biologically validated using external datasets. Our study highlights the clinical and biological relevance of proteomics in NEC patients. This study demonstrates key pathways involved in NEC pathogenesis and establishes DIA mass spectrometry as a powerful and noninvasive tool for evaluating and predicting NEC formation and progression.

**Graphical Abstract:**

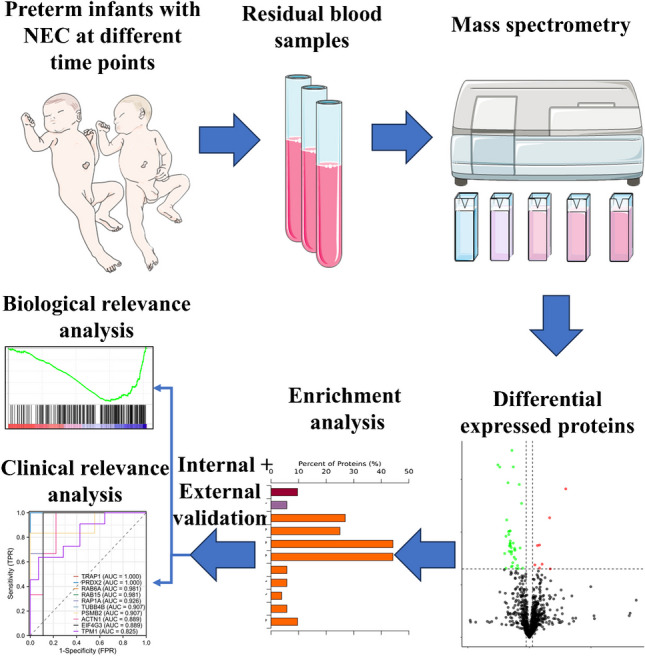

**Supplementary Information:**

The online version contains supplementary material available at 10.1007/s00216-024-05637-7.

## Introduction

Neonatal necrotizing enterocolitis (NEC) is a devastating disease with high mortality, and approximately 20–50% of patients necessitate surgical intervention [1–6]. It primarily affects premature and very low birth weight infants, typically presenting with symptoms such as vomiting, abdominal distension, and bloody stool [3]. Preterm infants weighing less than 1500 g are reported to have an incidence of NEC ranging from 2 to 7%, with a morality rate of 20 to 30%. Despite this, diagnostic and therapeutic options remain limited [2]. Current understanding suggests that factors such as immature intestinal development, formula feeding, dysbiosis, inflammation, ischemia, and necrosis contribute to the risk of NEC, yet its precise pathogenesis remains controversial [7]. NEC is known to progress rapidly, sometimes requiring surgery intervention; however, postoperative complications, including intestinal stenosis, short bowel syndrome, and neurodevelopmental retardation, must be carefully managed [3].

The evaluation and prediction of NEC are particularly challenging due to its diagnosis largely relying on clinical and basic radiologic features, which may overlap with other diseases [8]. Previous studies have sought to improve the evaluation and prediction of NEC, identifying potential biomarkers such as apoC2 and serum amyloid A [9]. For biomarkers to be clinically useful, they must be detectable in a noninvasive manner and applicable to routine clinical practice. Early detection of NEC before progression to a severe stage would enable preventive measures (e.g., probiotics administration) to be implemented [10].

Data-independent acquisition mass spectrometry (DIA MS) is a high-throughput technology that has been widely applied across various fields [11–13]. Recent studies have highlighted the potential of emerging biomarkers for the prediction and early diagnosis of NEC within the context of proteomics [9]. Notably, a Canadian group has recently reported one DIA MS study to analyze stool samples from NEC infants, presenting a promising approach for identifying NEC biomarkers [8]. However, stool samples are prone to contamination and difficult to preserve. In addition, blood transcriptomics has been explored as a surrogate biomarker for intestinal changes, owing to its capacity to reflect the systemic inflammatory response associated with NEC [14], yet transcriptomics often falls short of capturing the complete biological complexity of NEC, as messenger RNA (mRNA) levels do not always correlate with protein abundance or activity, which are crucial in understanding the disease’s pathophysiology [15].

To address these challenges, we employed DIA MS to compare differentially expressed proteins (DEPs) in plasma samples from NEC infants at different time points, with the goal of identifying potential plasma biomarkers for the evaluation and prediction of NEC formation and progression. Our study aims to facilitate a deeper understanding of the pathogenesis of NEC.

## Materials and methods

### Ethics declaration

The study was approved by the Ethics Committee of Shanghai Children’s Hospital (Approval No. 2019R083-E01) in accordance with the principles of the Declaration of Helsinki. Written informed consent to participate in this study was obtained from the legal guardians or next of kin of the participants. Patient identities and privacy were protected and anonymized throughout the study.

### Patients and specimens

The study included preterm neonates diagnosed with NEC based on clinical, radiological, and/or histopathological evidence that met Bell’s modified criteria. Exclusion criteria included non-NEC-evoked infection, hypoxia, postmaturity, and other congenital major anomalies according to established reports [1, 7, 10]. Eleven self-matched NEC infants were included, with the following time points: pre-NEC onset, NEC onset, NEC at week 1, and NEC at week 2. Specifically, we prospectively collected leftover blood samples from preterm infants during each routine examination, typically conducted weekly. Pre-NEC onset samples were prospectively collected from preterm infants fed by formula milk based on previous reports [16, 17]. Blood specimens were collected from hospitalized neonates in the neonatal intensive care unit (NICU) of Shanghai Children’s Hospital from April 1, 2019, to December 31, 2021. Blood samples were harvested in tubes with ethylenediaminetetraacetic acid (EDTA) and equilibrated under non-shaking conditions in a 4 °C refrigerator overnight. The plasma part was then separated and centrifuged at 12,000 × *g* for 30 min at 4 °C, and the supernatant was collected, aliquoted, and stored at − 80 °C.

### Sample preparation, quality control, and protein digestion

Frozen samples were thawed on ice and transferred to 1.5-mL centrifuge tubes. An appropriate amount of DB protein lysis buffer (8 M urea, 100 mM triethylammonium bicarbonate (TEAB), pH = 8.5) was added, followed by vortex and centrifugation at 12,000 × *g* for 15 min at 4 °C. The supernatant was transferred, and 10 mM dithiothreitol (DTT) was added at 56 °C for 1 h. The solution was then mixed with IAM at room temperature in the dark for 1 h.

Protein concentrations were measured using the Bradford Protein Assay Kit (Thermo Fisher, #23200) according to the manufacturer’s instructions. Denatured proteins (20 µg/lane) were separated by sodium dodecyl sulfate‒polyacrylamide gel electrophoresis (SDS‒PAGE) on 12% acrylamide gels. The running conditions were 80 V for 20 min for the stacking gel and 120 V for 90 min for the separating gel. The protein gels were stained using Coomassie brilliant blue R-250 dye (Thermo Fisher, #20278).

Samples that met the quality control (QC) criteria were transferred to new tubes, and DB protein lysis buffer (8 M urea, 100 mM TEAB, pH = 8.5) was added up to 100 µL. Trypsin and 100 mM TEAB buffer solution were added to the samples and incubated for 4 h at 37 °C, followed by the addition of trypsin and CaCl_2_ for overnight incubation. Formic acid was used to adjust the pH to less than 3. The supernatant was then collected by centrifugation at 12,000 × *g* for 5 min and transferred to a C18 desalting column. The digested samples were washed using cleaning solution (0.1% formic acid and 3% acetonitrile), eluted using elution buffer (0.1% formic acid and 70% acetonitrile), and lyophilized.

### Data-dependent acquisition spectral library construction

#### Separation of fractions

Mobile phases A (2% acetonitrile, adjusted pH to 10.0 using ammonium hydroxide) and B (98% acetonitrile, adjusted pH to 10.0 using ammonium hydroxide) were used to develop a gradient elution. The lyophilized powder was dissolved in solution A and centrifuged at 12,000 × *g* for 10 min at room temperature. The sample was fractionated using a C18 column (Waters BEH C18, 4.6 mm × 250 mm, 5 µm) on a Rigol L3000 high-performance liquid chromatography (HPLC) system, and the column oven was set at 45 °C. The details of the elution gradient are shown in Supplementary Table 1. The eluates were monitored at UV 214 nm, collected for a tube per minute, and combined into 5 fractions. All fractions were dried under vacuum and then reconstituted in 0.1% (v/v) formic acid (FA) in water.

#### LC-MS/MS analysis for data-dependent acquisition mode

For transition library construction, shotgun proteomics analyses were performed using an Evosep One UHPLC system (Evosep) coupled with a Q Exactive™ HF-X mass spectrometer (Thermo Fisher) operating in the data-dependent acquisition (DDA) mode. A half sample containing 4 µg fraction supernatant and 0.8 µl iRT reagent was separated in a homemade analytical column (15 cm × 150 µm, 1.9 µm) using a setup that allowed analysis of 30 samples per day. The separated peptides were analyzed by a Q Exactive™ HF-X mass spectrometer (Thermo Fisher) with an ion source of Nanospray Flex™ (ESI), a spray voltage of 2.1 kV, and an ion transport capillary temperature of 320 °C. The full scan range was from m/z 350 to 1500 with a resolution of 120,000 (at m/z 200), an automatic gain control (AGC) target value of 3 × 106, and a maximum ion injection time of 80 ms. The top 40 precursors with the highest abundance in the full scan were selected and fragmented by higher-energy collisional dissociation (HCD) and analyzed in MS/MS, where the resolution was 15,000 (at m/z 200), the AGC target value was 5 × 104, the maximum ion injection time was 45 ms, the normalized collision energy was 27%, the intensity threshold was 1.1 × 104, and the dynamic exclusion parameter was 20 s. The raw data of MS detection were named “.raw” and used to construct the DDA spectrum library.

### LC-MS/MS analysis–DIA mode

Mobile phases A (0.1% FA in H_2_O) and B (0.1% FA in 80% acetonitrile) were used to develop a gradient elution. A half sample containing 4 µg fraction supernatant and 0.8 µL iRT reagent was injected into the Evosep One UHPLC system (Evosep) coupled with an Orbitrap Q Exactive™ HF-X mass spectrometer (Thermo Fisher) operating in the DIA mode with a spray voltage of 2.1 kV, Nanospray Flex™ (ESI), and a capillary temperature of 320 ℃. For DIA acquisition, the m/z range covered from 350 to 1500. MS1 resolution was set to 60,000 (at m/z 200), full-scan AGC target value was 5 × 105, and the maximum ion injection time was 20 ms. Peptides were fragmented by HCD in MS2, in which resolution was set to 30,000 (at 200 m/z), AGC target value was 1 × 106, and normalized collision energy was 27%. The details of DIA mode scan are shown in Supplementary Table 2.

### Identification and quantification of proteins

Proteome Discoverer 2.2 (PD2.2; Thermo Fisher) software was used to search the data in DDA scanning mode. To be specific, the search parameters were set as follows: mass tolerance for precursor ion was 10 ppm, and mass tolerance for product ion was 0.02 Da. Carbamidomethyl was specified as fixed modifications. Oxidation of methionine was specified as dynamic modification. Acetylation was specified as N-terminal modification in PD2.2. A maximum of two missed cleavage sites were allowed.

The analysis was further modified using PD2.2 to ensure accurate results: (1) reliable peptides characterized by peptide spectrum matches (PSMs) with more than 99% confidence and (2) reliable proteins characterized by proteins containing at least one unique peptide. Only peptides and proteins with false discovery rate (FDR) ≤ 0.01 were retained.

Data extracted from PD2.2 were subsequently imported into Spectronaut (version 14.0, Biognosys) software to generate the spectrum library. A “Target List” was generated by selecting the qualified peptide segments and subions from the spectrum. Ion pair chromatographic peaks were extracted based on the imported DIA data and “Target List” to perform subion matching and peak area calculation, enabling simultaneous qualitative and quantitative analysis of peptide segments. Retention time correction was applied using the iRT kit (Biognosys) according to the manufacturer’s instructions. The cutoff of the *q* value of the precursor ion was set at 0.01.

### Protein-to-protein interaction network and correlation matrix

The STRING database (http://string-db.org/) was used to construct a protein-to-protein interaction (PPI) network for the DEPs with a minimum interaction score threshold of 0.15. The network was analyzed and visualized using Cytoscape (version 3.8.2) with the CytoHubba plugin, which identified hub genes. Additionally, gene–gene correlations were assessed using the Pearson correlation coefficient and visualized using the ggplot2 R package.

### Public data acquisition

For the external validation datasets, we retrieved the NEC proteomics dataset of serum (Mackay S. et al. [18]) and RNA microarray datasets of NEC-conditioned bowel tissues (GSE46619) and cells (GSE62208).

### Clinical assessment of the protein or gene in NEC

The prognostic value of the protein or gene was assessed using binary classifier receiver operating characteristic (ROC) curves, implemented with the “pROC” and “ggplot2” packages in R. The area under the curve (AUC) was calculated correspondingly.

### Gene set enrichment analysis

Gene set enrichment analysis (GSEA) software (version 4.3.2) was used to explore differences in gene expression profiles between the untreated epithelial cells and NEC-conditioned epithelial cells, as well as between NEC-conditioned bowel tissues and NEC-induced perforated bowel tissues. The analysis focused on biological process (BP), cell component (CC), and molecular function (MF) within Gene Ontology (GO) gene sets (10,461 sets, v7.5), Kyoto Encyclopedia of Genes and Genomes (KEGG) gene sets (805 sets, v7.5), Hallmark gene sets (50 sets, v7.5), HPO gene sets (55,653 sets, v7.5), and Wiki pathways gene sets (830 sets, v7.5) across diverse datasets. Statistical significance was determined by a normalized *P* (NOM *P*) value < 0.05 or an FDR < 0.25.

### Statistical analyses and functional study

Quantitative protein data were analyzed using the *t* test, and the significant DEPs between the NEC group and the control group were selected based on the criterion |fold change| > 1.2 with *P* < 0.05.

GO and IPR functions were annotated using InterProScan software, in conjunction with the Pfam, PRINTS, ProDom, SMART, ProSite, and PANTHER databases. Cluster of orthologous groups (COG) and KEGG analyses were performed to investigate the functional pathways of the identified proteins. The DEPs were analyzed and visualized using a volcano map, clustered heatmap, and enrichment plot. Some of data analysis and graphical presentations were generated using R software v4.2.1, GraphPad Prism v9.2.0, and GSEA v4.3.2. Unless noted otherwise in the specific methods previously mentioned, statistical significance was determined based on a *P* value threshold of < 0.05.

## Results

### Biosynthesis of protein and heme is inhibited during early stage of NEC formation

**Fig. 1:**
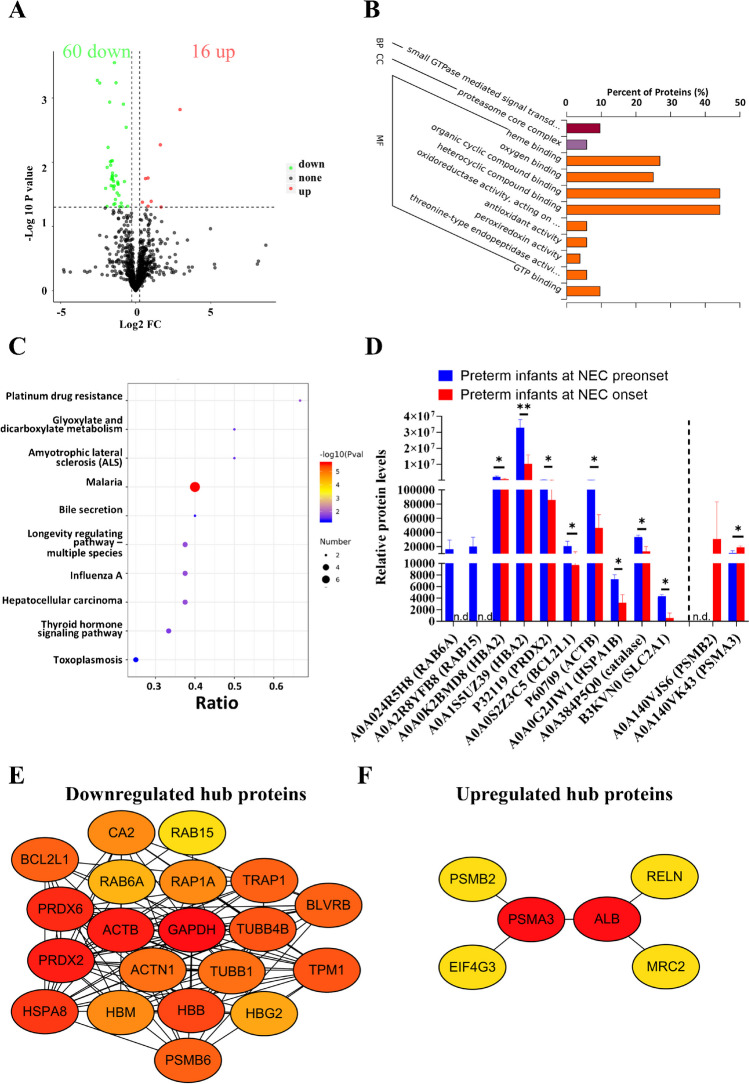
Biosynthetic processes of proteins and heme are inhibited in the early stage of neonatal necrotizing enterocolitis (NEC) formation. **A** Volcano plot showing the differentially expressed proteins (DEPs) between the preterm infants at NEC onset and pre-NEC onset. Gene Ontology (GO) (**B**) and Kyoto Encyclopedia of Genes and Genomes (KEGG) (**C**) analyses showing the enriched items between the two groups. **D** Box plot showing the representative differential proteins between the two groups. Protein-to-protein interaction (PPI) network showing the hub downregulated (**E**) and upregulated (**F**) DEPs using the degree algorithm. BP, biological process; CC, cell component; FC, fold change; MF, molecular function; n.d., not detected. *P < 0.05; **P < 0.01

A total of 76 DEPs were identified between preterm infants at NEC onset and pre-NEC onset by DIA MS analysis (Fig. 1A), with the top 10 DEPs represented in Table 1. These altered proteins were enriched in GO annotations, including one BP, one CC, nine MF items (Fig. 1B), and ten KEGG pathways (Fig. 1C). The GO annotations primarily highlighted a reduction in “small GTPase-mediated signal transduction.” In addition, the observed decreases in “heterocyclic compound binding” and “organic cyclic compound binding” might contribute to the “diminished GTP binding” and “heme/oxygen binding” activities (Fig. 1B). These findings were supported by the decreased expression of A0A024R5H8 (RAB6A), A0A2R8YFB8 (RAB15), and A0A0K2BMD8/A0A1S5UZ39 (HBA2) (Fig. 1D). In addition, some GO-MF-enriched items, such as “oxidoreductase activity, acting on peroxide as acceptor,” showed reduced antioxidant activity (Fig. 1B), evidenced by the decreased expression of P32119 (PRDX2) (Fig. 1D). Moreover, our GO-CC and GO-MF analyses showed potential proteolysis, indicated by the enhanced “proteosome core complex” and “threonine-type endopeptidase activity” (Fig. 1B), as evidenced by the increased expression of A0A140VJS6 (PSMB2) and A0A140VK43 (PSMA3) (Fig. 1D).

**Table 1:** List of the top 10 DEPs during the early stage of NEC

Protein code	Protein name
Upregulated proteins	
A0A140VJS6	Proteasome subunit beta
A2N2W8	VL6 protein (fragment)
Q9UBG0	C-type mannose receptor 2
B2RBF5	cDNA, FLJ95483, highly similar to *Homo sapiens *di-*N*-acetyl-chitobiase (CTBS), mRNA
A0A0G2JMY9	Leukocyte immunoglobulin-like receptor subfamily A member 3
A0A0F7T983	IGHV1-2 protein (fragment)
Q96JD2	Amyloid lambda 6 light-chain variable region NEG (fragment)
A0A024RDA6	Insulin-like growth factor–binding protein 7, isoform CRA_a (fragment)
J3KQ66	Reelin
S6AWF0	IgG H chain
Downregulated proteins	
L7UUZ7	Integrin beta
P68371	Tubulin beta-4B chain
A0A2R8YFB8	Ras-related protein Rab-15
A0A024R5H8	RAB6A, member RAS oncogene family, isoform CRA_b
Q6FIG4	RAB1B protein
B4DTA6	cDNA FLJ57794, moderately similar to Ras-related protein Rab-35
A0A140VJY2	Testicular tissue protein Li 209
A8KAH9	RAP1A, member of RAS oncogene family
P00918	Carbonic anhydrase 2
A0A024R694	Actinin, alpha 1, isoform CRA_a

The top ten DEPs between preterm infants at NEC onset and pre-NEC onset are shown

Consistent with GO annotations, the KEGG pathway “malaria” was enriched (Fig. 1C), accompanied by a decreased level of HBA2 (Fig. 1D), suggesting reduced heme/oxygen binding. In addition, KEGG analysis indicated the involvement of apoptotic pathways, including “platinum drug resistance,” “hepatocellular carcinoma,” “thyroid hormone signaling pathway,” and “amyotrophic lateral sclerosis” (Fig. 1C), as highlighted by the decreased expression of A0A0S2Z3C5 (BCL2L1) and P60709 (ACTB) (Fig. 1D). Decreased levels of A0A0G2JIW1 (HSPA1B) and A0A384P5Q0 (catalase) along with enriched KEGG pathways including “longevity regulating pathway - multiple species,” “influenza A,” “glyoxylate and dicarboxylate metabolism,” and “toxoplasmosis” suggest vulnerabilities to aging and oxidative stress (Fig. 1C, D). Interestingly, the KEGG analysis implicated bile secretion in the decreased level of B3KVN0 (SLC12A2) (Fig. 1D), suggesting attenuated bile secretion during NEC formation, consistent with our previous reports [19].

Furthermore, we integrated significantly downregulated and upregulated proteins into a hub protein network associated with the early stage of NEC formation (Fig. 1E, F). Downregulated proteins such as HBB, TUBB4B, and ACTB exhibited positive correlations with each other, while they were negatively correlated with the upregulated proteins such as PSMA3, PSMB2, and MRC2 (Supplementary Fig. 1A).

**Fig. 2:**
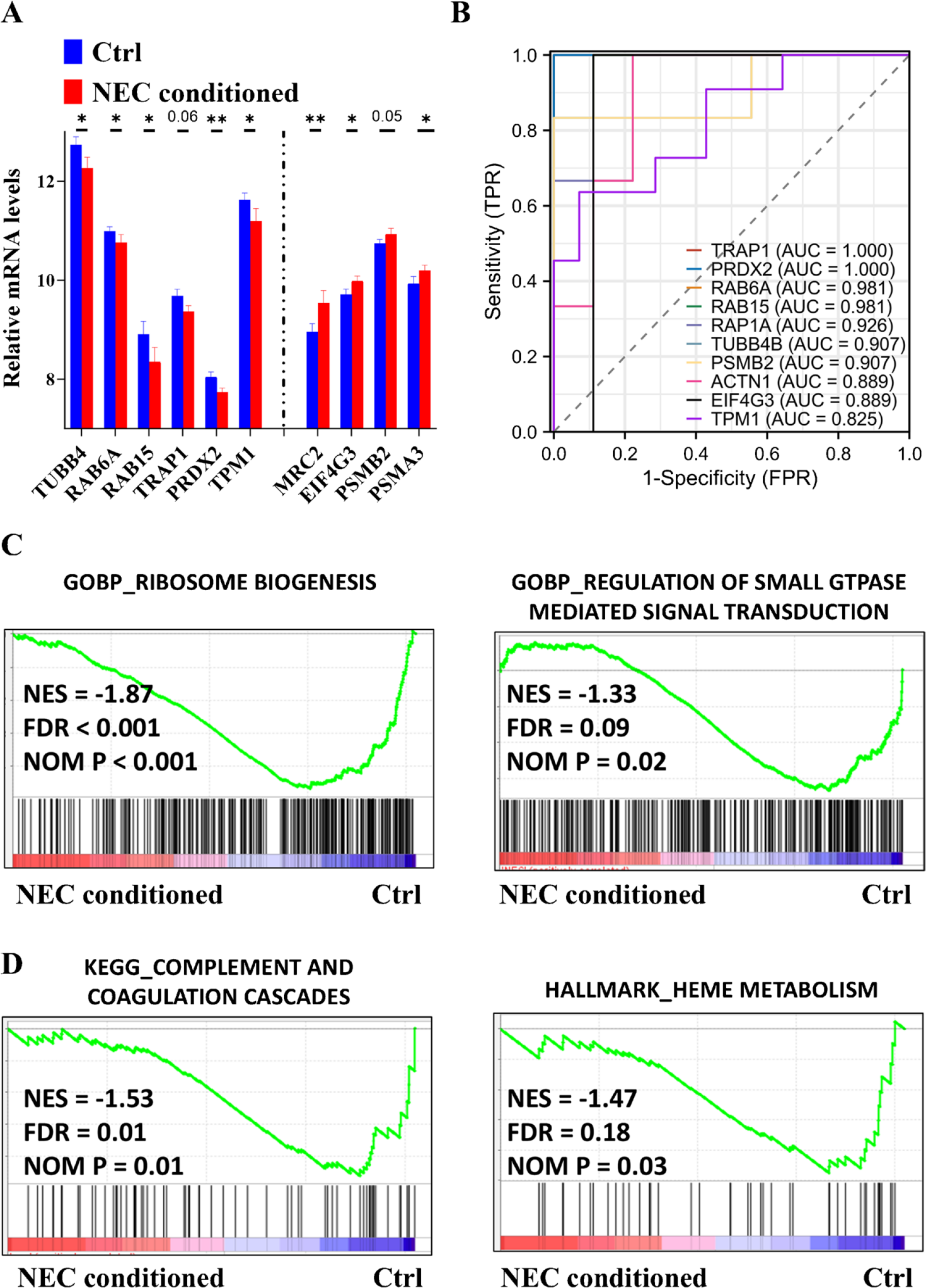
Inhibited proteins and heme biosynthesis are clinically and biologically validated using external datasets. **A** Box plots illustrating the validation of altered mRNAs (GSE46619) during the early stages of neonatal necrotizing enterocolitis (NEC) formation. **B** Receiver operating characteristic (ROC) curves generated by integrating internal and external datasets (GSE46619 and Mackay S. et al. [18]) to assess the early stage of NEC formation. Representative enriched gene sets for inhibited protein biosynthesis (**C**) and suppressed heme production (**D**) were identified using cell data (GSE62208), corresponding to the early stage of NEC formation. AUC, area under the curve; BP, biological process; Ctrl, control; FDR, false discovery rate; FPR, false positive rate; GO, Gene Ontology; NES, normalized enrichment score; NOM, normalized; TPR, true positive rate. *P < 0.05; **P < 0.01

Subsequently, we utilized several external datasets, including NEC serum, tissues, and NEC-conditioned bowel epithelial cells, to validate our findings. Little data from the proteomic analysis showed slight changes in GAPDH and PRDX6 between non-NEC and NEC-conditioned serum (Supplementary Fig. 1B); however, the majority of our findings were corroborated by RNA microarray data (Fig. 2A). Moreover, by integrating internal and external datasets, we demonstrated the prognostic value of protein markers during the early stage of NEC, with most AUC values exceeding 0.80 (Fig. 2B). Last, we validated the biological relevance using NEC-conditioned cell data, which revealed inhibited protein (Fig. 2C) and heme (Fig. 2D) biogenesis. Together, our results suggest a protein network characterized by dysregulated hemostasis including suppressed wound healing and coagulation and increased proteolysis during the early stage of NEC formation.

### Nitrogen metabolism and purine nucleotide biosynthesis are suppressed during the late stage of NEC formation

**Fig. 3:**
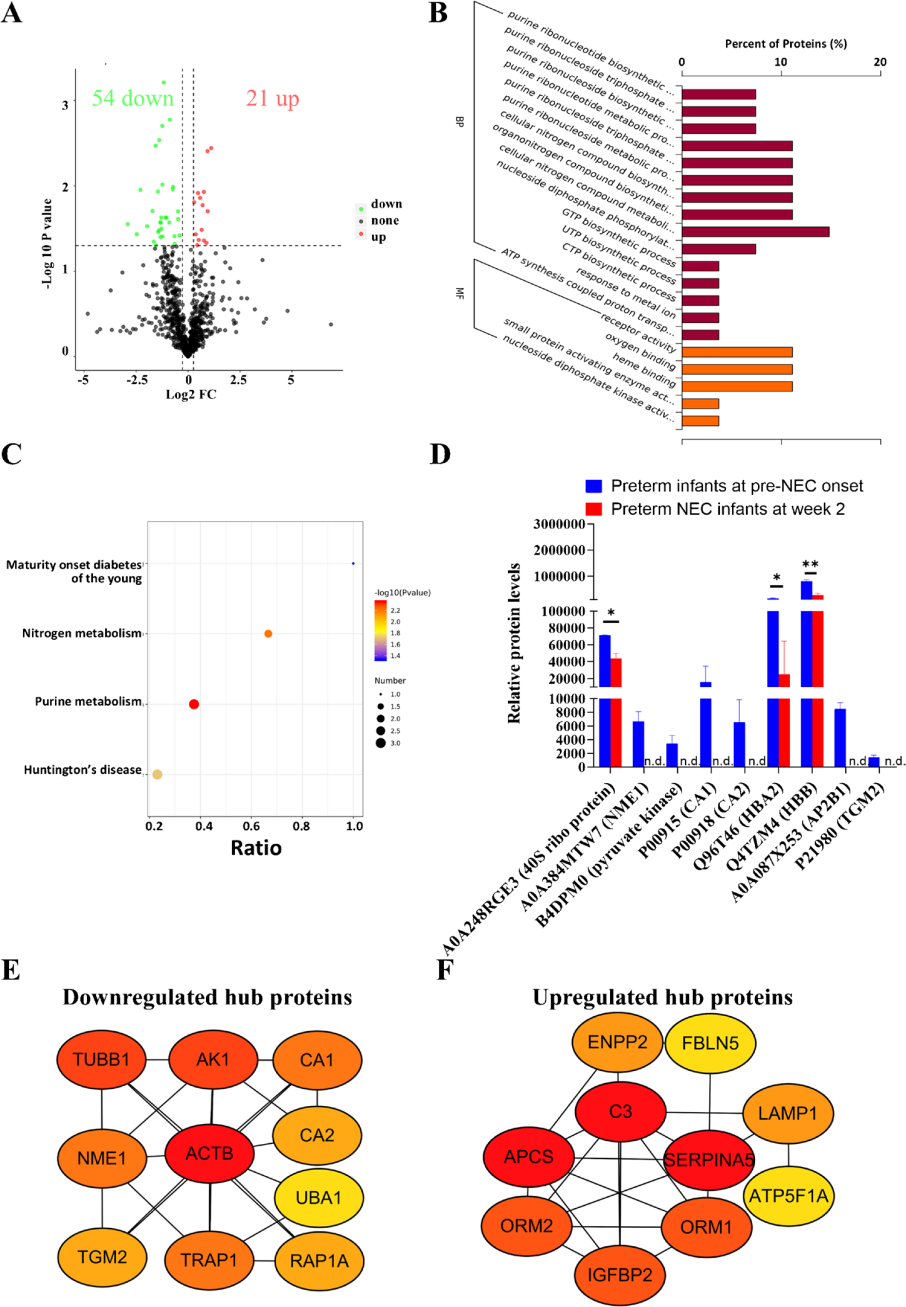
Nitrogen and purine nucleotide biosynthesis are inhibited during the late stage of neonatal necrotizing enterocolitis (NEC) formation. **A** Volcano plot showing the differentially expressed proteins (DEPs) between preterm NEC infants at week 2 and pre-NEC onset infants. Gene Ontology (GO) (**B**) and Kyoto Encyclopedia of Genes and Genomes (KEGG) (**C**) analyses showing the enriched items between the two groups. **D** Box plot showing the representative differential proteins between the two groups. Protein-to-protein interaction (PPI) network showing the hub downregulated (**E**) and upregulated (**F**) DEPs using the degree algorithm. BP, biological process; FC, fold change; MF, molecular function; n.d., not detected. *P < 0.05; **P < 0.01

During the later stage of NEC formation, 75 DEPs were identified in the plasma of preterm NEC infants at week 2 compared to the pre-NEC stage (Fig. 3A), with the top 10 DEPs presented in Table 2. GO analysis enriched 15 BP and 5 MF items (Fig. 3B), and KEGG pathway analysis identified 4 items (Fig. 3C). Notably, GO analysis highlighted reductions in “cellular nitrogen compound metabolism” and “purine ribonucleoside metabolic process” (Fig. 3B), evidenced by decreased levels of specific proteins such as A0A248RGE3 (40S ribosomal protein) and B4DPM0 (pyruvate kinase) (Fig. 3D). The KEGG analysis supported this, showing reduced nitrogen and purine metabolism pathways (Fig. 3C), aligned with decreased levels of P00915 (CA1) and P00918 (CA2) (Fig. 3D).

**Table 2:** List of the top ten DEPs during the late stage of NEC

Code in library	Protein name
Upregulated proteins	
B4DI57	cDNA FLJ54111, highly similar to serotransferrin
A0A087WTY6	Neuroblastoma suppressor of tumorigenicity 1
A0A024RDY3	Lysosomal-associated membrane protein 1, isoform CRA_a
B2RBF5	cDNA, FLJ95483, highly similar to *Homo sapiens*, di-*N*-acetyl-chitobiase (CTBS), mRNA
M0R1Q1	Complement C3 (fragment)
A0A140VJS6	Proteasome subunit beta
A0A024RDA6	Insulin-like growth factor–binding protein 7, isoform CRA_a (fragment)
P02743	Serum amyloid P-component
A0A024R6G3	Fibulin 5, isoform CRA_b
P02763	Alpha-1-acid glycoprotein 1
Downregulated proteins	
A0A193CHS1	10E8 light-chain variable region (fragment)
Q6PIK1	IGL@ protein
A0A0X9UWJ6	MS-B1 light-chain variable region (fragment)
A0A2U8J9C0	Ig heavy-chain variable region (fragment)
P00915	Carbonic anhydrase 1
A0A2U8J975	Ig heavy-chain variable region (fragment)
A0A0X9V9C4	GCT-A8 heavy-chain variable region (fragment)
A0A140VJY2	Testicular tissue protein Li 209
A0A2U8J8T6	Ig heavy-chain variable region (fragment)
A0A087X253	AP complex subunit beta

The top ten DEPs between preterm NEC infants at week 2 and pre-NEC onset are shown

Furthermore, hub protein networks showed positive correlations among downregulated proteins such as TRAP1 and AK1, while these proteins negatively correlated with upregulated proteins such as C3 and ORM1 (Fig. 3E, F and Supplementary Fig. 2A).

**Fig. 4:**
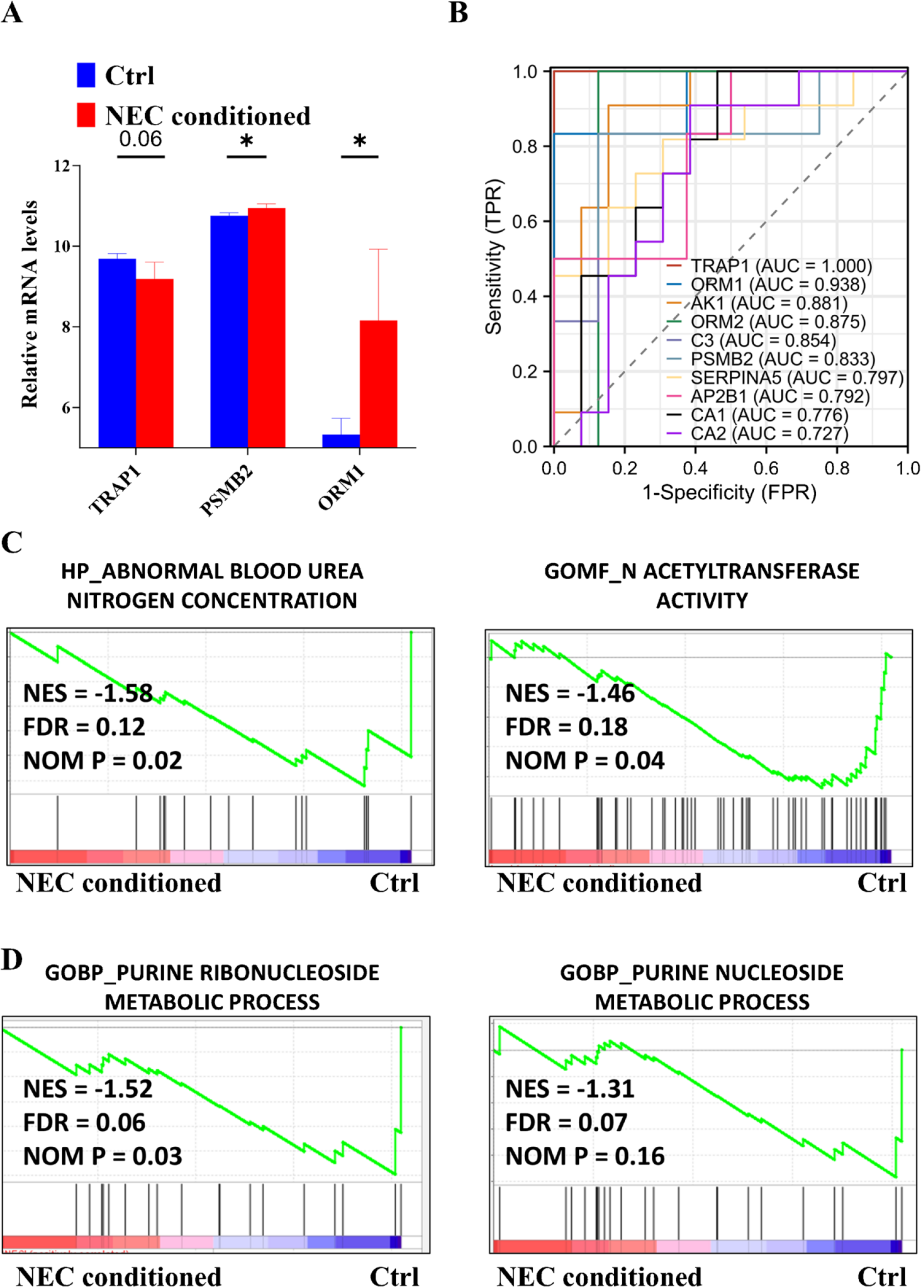
Reductions in nitrogen and purine nucleotide biosynthesis are validated both clinically and biologically using external datasets. **A** Box plots illustrating the validation of altered mRNAs (GSE46619) during long-term neonatal necrotizing enterocolitis (NEC) formation. **B** Receiver operating characteristic (ROC) curves generated by integrating internal and external datasets (GSE46619 and Mackay S. et al. [18]) to assess long-term NEC formation. Representative enriched gene sets for inhibited nitrogen production (**C**) and suppressed purine nucleotide biosynthesis (**D**) were identified using cell data (GSE62208), corresponding to the late stage of NEC formation. AUC, area under the curve; BP, biological process; Ctrl, control; FDR, false discovery rate; FPR, false positive rate; GO, Gene Ontology; NES, normalized enrichment score; NOM, normalized; TPR, true positive rate. *P < 0.05

Next, external proteomic data showed significant decrease levels in CA1, AK1, and NME1 between non-NEC and NEC-conditioned serum (Supplementary Fig. 2B), partially supported by RNA microarray data (Fig. 4A). Moreover, integration of internal and external datasets demonstrated the prognostic value of protein markers during late-stage NEC formation, with most AUC values over 0.75 (Fig. 4B). Finally, NEC-conditioned cell data validated aberrant nitrogen metabolism (Fig. 4C) and inhibited purine nucleotide biogenesis (Fig. 4D). Collectively, these results unveil a protein network marked by attenuated nitrogen and purine biosynthesis during late-stage NEC formation.

### Mixed extracellular activities and immune response are implicated during the short-term progression of NEC

**Fig. 5:**
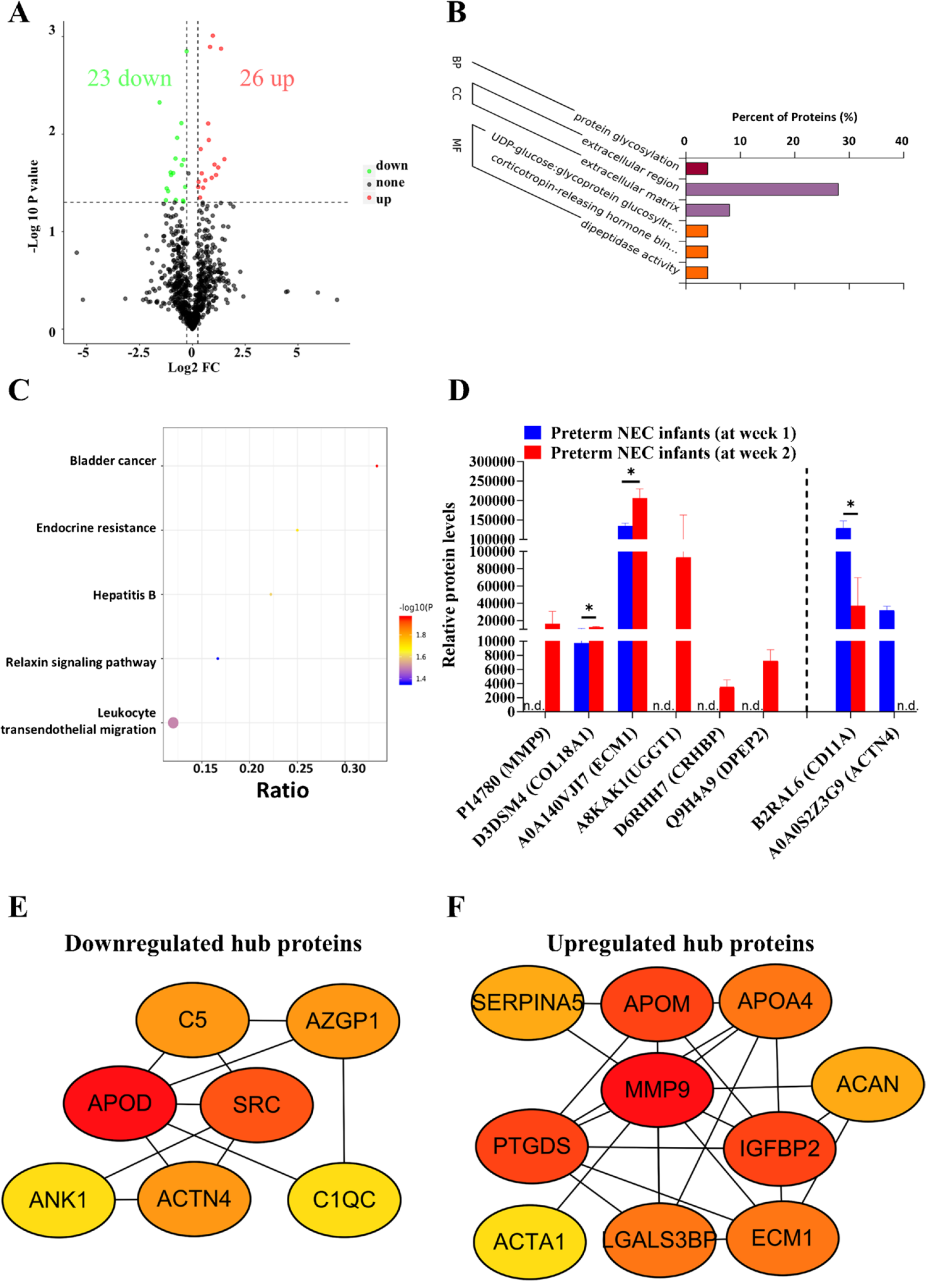
Extracellular reconstruction and altered immune response are implicated during the short-term progression of neonatal necrotizing enterocolitis (NEC). **A** Volcano plot showing the differentially expressed proteins (DEPs) between preterm NEC infants at week 2 and NEC infants at week 1. Gene Ontology (GO) (**B**) and Kyoto Encyclopedia of Genes and Genomes (KEGG) (**C**) analyses showing the enriched items between the two groups. **D** Box plot showing the representative differential proteins between the two groups. Protein-to-protein interaction (PPI) network showing the hub downregulated (**E**) and upregulated (**F**) DEPs using the degree algorithm during the short-term of NEC progression. BP, biological process; CC, cell component; FC, fold change; MF, molecular function; n.d., not detected. *P < 0.05

During the short-term progression of NEC, 49 DEPs were identified in the plasma of preterm NEC infants at week 2 compared to week 1 (Fig. 5A), with the top 10 DEPs listed in Table 3. GO analysis revealed enrichment in one BP, two CC, and three MF items (Fig. 5B), and KEGG analysis identified five enriched pathways (Fig. 5C). Among the GO categories, the CC items exhibited the highest protein ratio values concerning “extracellular region” and “extracellular matrix (ECM)” (Fig. 5B), represented by the increased protein levels of P14780 (MMP9), D3DSM4 (COL18A1), and A0A140VJI7 (ECM1), underscoring the importance of extracellular activities during short-term development of NEC (Fig. 5D). Other items including protein glycosylation, corticotropin-releasing hormone (CRH) binding, and dipeptidase activity (Fig. 5B), as evidenced by increased levels of A8KAK1 (UGGT1), D6RHH7 (CRHBP), and Q9H4A9 (DPEP2) (Fig. 5D), might be indirectly related to extracellular reconstruction and wound healing under inflammatory stress [20–22]. In addition, KEGG pathway enrichment analysis identified pathological conditions related to cancer, endocrine disorder, infection, and relaxin signaling (Fig. 5C), characterized by increased MMP9 level (Fig. 5D). Interestingly, our KEGG results suggest potential dual regulation of leukocyte trans-endothelial migration (Fig. 5C), as evidenced by an increase level of MMP9 and decreased levels of B2RAL6 (highly similar to CD11A) and A0A0S2Z3G9 (ACTN4) (Fig. 5D).

**Table 3:** List of the top ten DEPs in terms of NEC short-term development

Protein code	Protein name
Upregulated proteins	
A8KAK1	cDNA FLJ77398, highly similar to *Homo sapiens* UDP-glucose ceramide glucosyltransferase-like 1, transcript variant 2, mRNA
Q86U17	Serpin A11
Q9UK54	Hemoglobin beta subunit variant (fragment)
P14780	Matrix metalloproteinase-9
Q9H4A9	Dipeptidase 2
Q6E0U4	Dermokine
D6RHH7	Corticotropin-releasing factor-binding protein
A8K7G6	cDNA FLJ75763, highly similar to *Homo sapiens* regenerating islet-derived 1 alpha (pancreatic stone protein, pancreatic thread protein) (REG1A), mRNA
A0A087WXS7	ATPase ASNA1
P68133	Actin, alpha skeletal muscle
Downregulated proteins	
A0A193CHS1	10E8 light-chain variable region (fragment)
A0A0S2Z3G9	Actinin alpha 4 isoform 1 (fragment)
B2R6A3	Na(+)/H(+) exchange regulatory cofactor NHE-RF
A6MW40	PKDREJ (fragment)
P12931	Proto-oncogene tyrosine–protein kinase Src
P60985	Keratinocyte differentiation–associated protein
E7EWH8	Putative hydroxypyruvate isomerase
P01602	Immunoglobulin kappa variables 1–5
B2RAL6	cDNA, FLJ94991, highly similar to *Homo sapiens* integrin, alpha L (antigen CD11A (p180), lymphocyte function–associated antigen 1 alpha polypeptide) (ITGAL), mRNA
Q5FWF9	IGL@ protein

The top ten DEPs between the preterm NEC infants at week 2 and at week 1 are shown

Furthermore, hub protein networks including downregulated and upregulated DEPs were constructed for the short-term progression of NEC (Fig. 5E, F), revealing that downregulated proteins such as ACTN4, SRC, and KRTDAP were positively correlated with each other, while these proteins were negatively correlated with upregulated proteins such as MMP9, SERPINA5, and ACAN (Supplementary Fig. 3A).

**Fig. 6:**
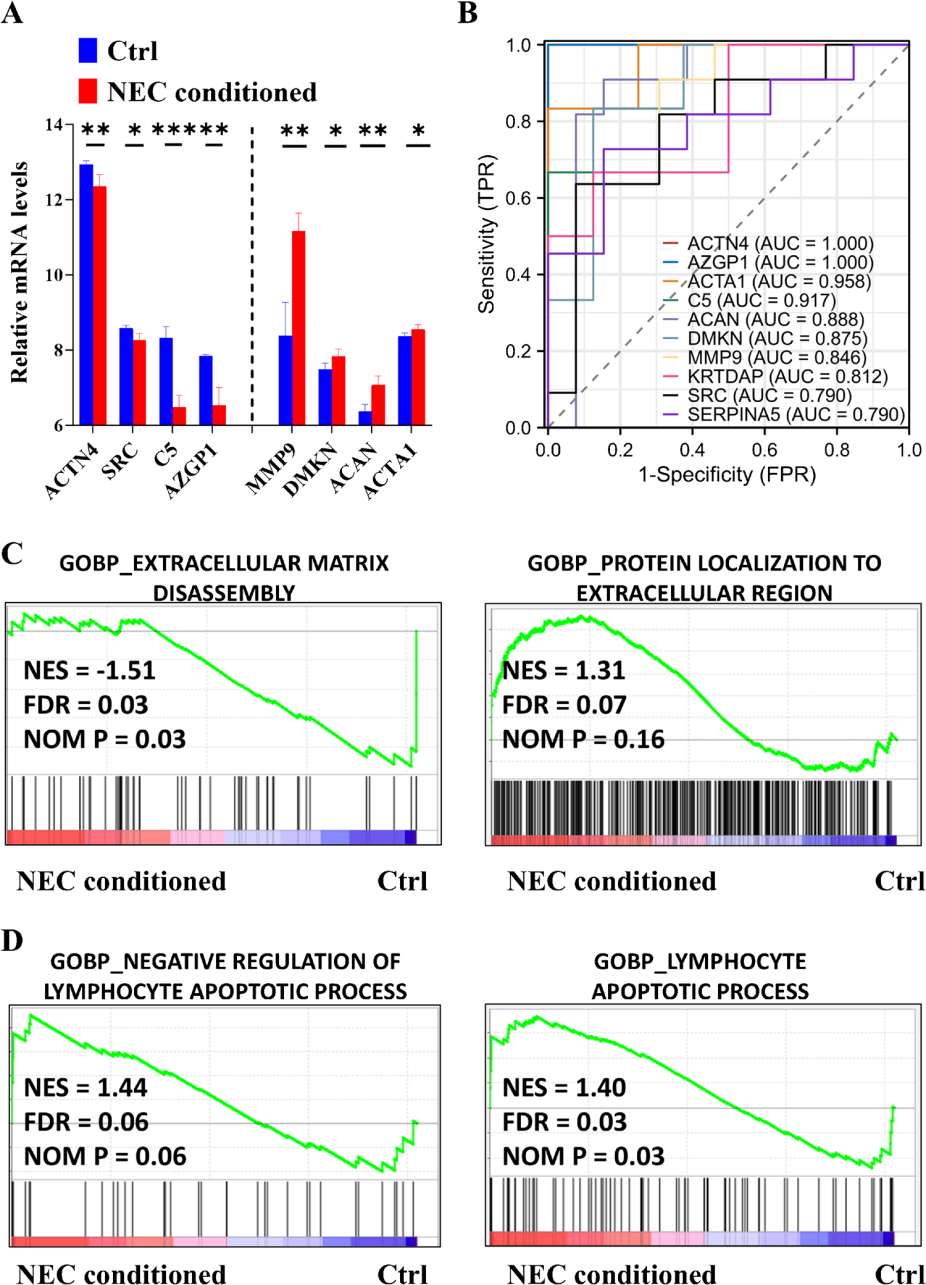
Reconstruction of extracellular matrix (ECM) during short-term development of neonatal necrotizing enterocolitis (NEC) is clinically and biologically validated using external datasets. **A** Box plots illustrating the validation of altered mRNAs (GSE46619) during short-term NEC progression. **B** Receiver operating characteristic (ROC) curves generated by integrating internal and external datasets (GSE46619 and Mackay S. et al. [18]) to assess short-term NEC progression. Representative enriched gene sets for ECM reconstruction (**C**) and altered lymphocyte apoptosis (**D**) were identified using cell data (GSE62208), corresponding to short-term NEC progression. AUC, area under the curve; BP, biological process; Ctrl: control; FDR, false discovery rate; FPR, false positive rate; GO, Gene Ontology; NES, normalized enrichment score; NOM, normalized; TPR, true positive rate. *P < 0.05; **P < 0.01; ***P < 0.001

Then, external datasets were analyzed to validate our findings. Data from the external proteomics showed a significant increase in A0A0S2Z3Y1 (LGALS3BP) (Supplementary Fig. 3B), and most of our results were validated by RNA microarray data (Fig. 6A). Furthermore, by integrating internal and external datasets, we demonstrated the prognostic value of these protein markers in the short-term progression of NEC, with most AUC values exceeding 0.80 (Fig. 6B). Finally, the biological relevance of these findings was confirmed using NEC-conditioned cell data, which revealed a reconstructed ECM (Fig. 6C) and mixed lymphocyte apoptotic processes (Fig. 6D). Together, these findings highlight a protein network characterized by the reconstruction of extracellular components and a mixed immune response during the short-term progression of NEC.

### Dysregulated B-cell immune response and reduced glycosaminoglycan metabolism are identified during the long-term progression of NEC

**Fig. 7:**
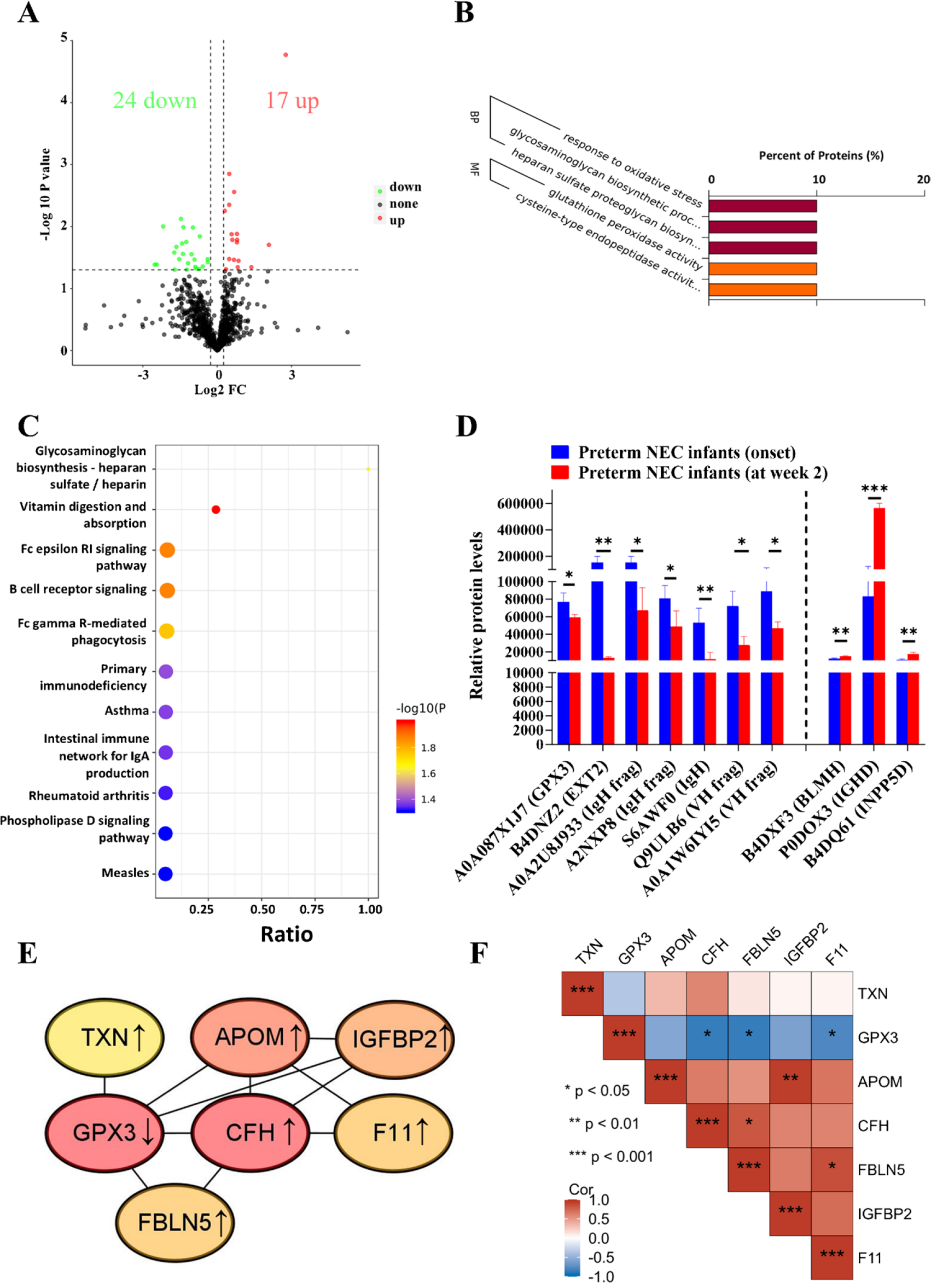
Dysregulated B-cell immune response and repressed glycosaminoglycan (GAG) biosynthesis are found in the long-term development of neonatal necrotizing enterocolitis (NEC). **A** Volcano plot showing the differentially expressed proteins (DEPs) between preterm NEC infants at week 2 and NEC infants at onset. Gene Ontology (GO) (**B**) and Kyoto Encyclopedia of Genes and Genomes (KEGG) (**C**) analyses showing the enriched items between the two groups. **D** Box plot showing the representative differential proteins between the two groups. **E** Protein-to-protein interaction (PPI) network showing the hub DEPs using the degree algorithm. **F** A matrix showing protein–protein correlations among hub proteins during the long-term NEC progression. BP, biological process; FC, fold change; MF, molecular function. *P < 0.05; **P < 0.01; ***P < 0.001

DIA MS analysis showed 41 DEPs between preterm infants with NEC at week 2 and at the onset of NEC (Fig. 7A), with the top 10 DEPs illustrated in Table 4. The GO annotations mainly highlighted reductions in “response to oxidative stress” and “glutathione peroxidase activity” (Fig. 7B), indicated by decreased A0A087X1J7 (GPX3) levels (Fig. 7D). In addition, there were decreases in “GAG biosynthetic process” and “heparan sulfate proteoglycan biosynthetic process” (Fig. 7B), supported by lower levels of B4DNZ2 (EXT2) (Fig. 7D).

**Table 4:** List of the top ten DEPs in terms of NEC long-term development

Protein code	Protein name
Upregulated proteins	
P0DOX3	Immunoglobulin delta heavy chain
Q6ZVX0	cDNA FLJ41981 fis, clone SMINT2011888, highly similar to protein Tro alpha 1 H, myeloma
Q86UX7	Fermitin family homolog 3
A0PJ79	MRPL1 protein (fragment)
Q59HB3	Apolipoprotein B variant (fragment)
A0A1U9X793	APOM
A0A2U8J8W3	Ig heavy-chain variable region (fragment)
O95445	Apolipoprotein M
B4DQ61	cDNA FLJ56795, highly similar to *Homo sapiens* inositol polyphosphate-5-phosphatase, 145 kDA (INPP5D), transcript variant 1, mRNA
P03951	Coagulation factor XI
Downregulated proteins	
A2JA16	Anti-mucin 1 light-chain variable region (fragment)
0A075B6S9	Immunoglobulin kappa variables 1–37 (non-functional) (fragment)
S6AWF0	IgG H chain
Q7Z2U7	Uncharacterized protein
Q7Z351	Uncharacterized protein DKFZp686N02209
A0A2U8J8K8	Ig heavy-chain variable region (fragment)
B0YIZ6	Cubilin variant 3
A2NWW3	VH-3 family (VH26)D/J protein (fragment)
A0A0C4DH25	Immunoglobulin kappa variable 3D-20
A0A075B7D0	Immunoglobulin heavy-chain variable 1/OR15-1 (non-functional) (fragment)

The top ten DEPs between preterm NEC infants at week 2 and at NEC onset are shown

KEGG pathway analysis highlighted impaired B-cell- and immunoglobulin-associated immune responses (Fig. 7C), including pathways such as “B cell receptor signaling pathway,” “Fc epsilon RI signaling pathway,” and “Fc gamma R-mediated phagocytosis.” These changes were reflected by decreased levels of A0A2U8J933 (IgH frag), A2NXP8 (IgH frag), S6AWF0 (IgH), Q9ULB6 (VH frag), and A0A1W6IYI5 (VH frag), and increased levels of P0DOX3 (IGHD) and B4DQ61 (INPP5D) (Fig. 7D). Moreover, a reduction in glycosaminoglycan (GAG) metabolism was supported by KEGG analysis (Fig. 7C), corroborated by a decrease level of B4DNZ2 (Fig. 7D).

Hub protein network analysis showed upregulated proteins (APOM, CFH, FBLN5, IGFBP2, and F11) positively correlated with each other and negatively correlated with the downregulated protein GPX3 during the long-term progression of NEC (Fig. 7E, F).

**Fig. 8:**
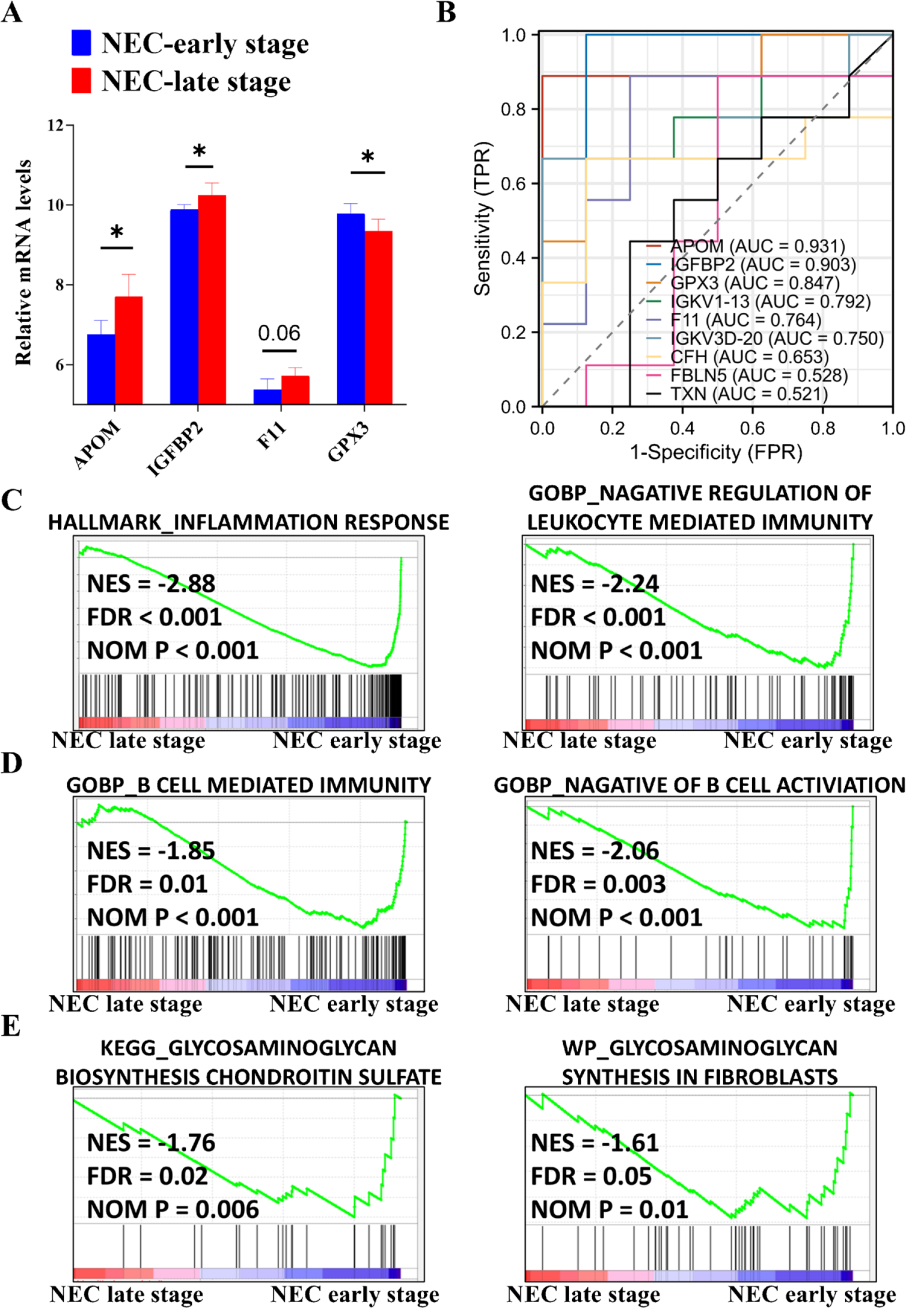
Dysregulated B-cell immune response and reduced glycosaminoglycan (GAG) in the long-term development of neonatal necrotizing enterocolitis (NEC) are clinically and biologically validated using external datasets. **A** Box plots illustrating the validation of altered mRNAs (GSE46619) during long-term NEC progression. **B** Receiver operating characteristic (ROC) curves generated by integrating internal and external datasets (GSE46619 and Mackay S. et al. [18]) to assess long-term NEC progression. Representative enriched gene sets for inflammation response (**C**), B-cell-mediated immune response (**D**), and GAG metabolism (**E**) were identified using cell data (GSE62208), corresponding to long-term NEC progression. AUC, area under the curve; BP, biological process; FDR, false discovery rate; FPR, false positive rate; GO: Gene Ontology; NES, normalized enrichment score; NOM, normalized; TPR, true positive rate. *P < 0.05

The majority of our results were corroborated by external RNA microarray data (Fig. 8A). By integrating both internal and external datasets, we revealed protein markers with most AUC over 0.75 (Fig. 8B). Eventually, the biological relevance of these findings was confirmed using external data from perforated NEC tissues, which demonstrated dysfunction in overall inflammatory responses (Fig. 8C) and B-cell immune responses (Fig. 8D), as well as a reduction in GAG metabolism (Fig. 8E) during NEC progression.

In summary, we identified a dysregulated immune response, particularly in B-cell-associated immune response, and a decrease in GAG metabolism as key factors in the development of NEC.

## Discussion

NEC is a predominantly premature infant disease and one of the most devastating conditions in the NICU [1, 3, 8]. In this study, we collected peripheral blood samples from NEC infants at different time points and extracted plasma protein to analyze DEPs using DIA technology. Our findings revealed a reduction in protein, heme, nitrogen, and purine nucleotide biosynthesis during NEC formation, as well as alterations in ECM, aberrant B-cell immune responses, and reduced GAG level during NEC progression. These results were validated both clinically and biologically using external datasets. Our work underscores the potential of DIA in the evaluation and prediction of NEC.

Over the past decade, emerging high-throughput technologies such as transcriptomics, proteomics, and metabolomics have been increasingly performed to identify biomarkers and predict NEC at an early stage [9]. Blood transcriptomics has been investigated as a surrogate biomarker for intestinal alterations due to its ability to mirror the inflammatory response linked to NEC [14]. However, transcriptomics frequently fails to fully capture the biological complexity of NEC, as mRNA levels do not consistently correlate with protein abundance or activity [15]. In addition, DDA technology has traditionally been the mainstay of proteomics studies. However, DDA is limited in its capacity to analyze samples with complex components [23]. In contrast, DIA is a novel, comprehensive, and data-independent acquisition technology that successively fragments all parent ions within a specific mass range and collect fragment ion information for protein qualitative and quantitative protein analysis [8, 23]. Various biological fluids and tissues, such as serum, plasma, urine, saliva, feces, and surgically removed tissues, can be used for proteomic profiling. Our work demonstrated the application of DIA in analyzing plasma samples from NEC infants.

The DEPs identified in the early phase of NEC indicated diminished protein and heme production during the onset of NEC. Small GTPase-mediated signal transduction, involving protein families such as Ras, Rab, and Rho GTP-binding proteins, plays a critical role in protein biosynthesis [24]. Specifically, the Rab family protein RAB6A controls vesicle transportation through the Golgi apparatus and RAB15 promotes cell proliferation [24–26]. Previous studies have also linked small GTPase-mediated signal transduction to heterocyclic compound binding, which facilitates GTP binding [27]. In addition, we observed increased levels of PSMB2 and PSMA3, indicative of enhanced proteolysis, which aligned with findings in various inflammatory diseases [28, 29]. Furthermore, an increase in apoptotic processes was observed, accompanied by decreased levels of BCL2L1 and ACTB, corresponding to previous reports [30, 31]. Organic cyclic compound binding, typically associated with the biosynthetic process of heme and hemoglobin, was also noted [32]. Also, classical inflammatory biomarkers in NEC such as IL-6 and TNF-α accelerate proteolysis [33, 34] and C-reactive protein shows an inverse relationship with hemoglobin [35]. Overall, our results could reflect clinical scenarios characterized by exacerbated intestinal injury due to impaired small-GTPase activity and increased proteolysis and apoptosis, and heme-positive stools caused by defective heme binding [36].

Moreover, we detected the decreased levels of PRDX2, HSPA1B, and catalase, suggesting a diminished antioxidative capacity in NEC, which was consistent with previous studies on necrotizing colitis or enterocolitis [37–39]. Interestingly, our findings aligned with one of our previous studies showing that activation of the farnesoid X–activated receptor stimulated bile secretion and prevented the formation of NEC (data submitted elsewhere), as reflected in the decreased levels of SLC12A2. Also, ileal bile acid–binding protein might be a NEC biomarker for intestinal epithelial cell damage [40]. These results might reflect clinical themes characterized by increased intestinal vulnerabilities due to impaired antioxidative capacity and bile secretion.

In the late phase of NEC occurrence, the DEPs indicated a reduction in nitrogen and purine nucleotides. It is well established that nitrogen is crucial for DNA synthesis and cell division [41]. A prior metabolomic study also linked plasma from NEC piglets to metabolites involved in purine nucleotide metabolism [42]. Additionally, DNA replication is intricately connected to purine and pyrimidine nucleotides, which promote cell growth [43]. Some alterations in the late phase mirrored those in the early phase, such as the reduction in heme binding. Interestingly, we found metabolic alterations related to glucose, evidenced by decreased levels of pyruvate kinase and TGM2. Huang et al. [44] proposed that pyruvate suppressed epithelial cell death in an ATP-independent manner, and Kumar et al. [45] suggested that TG2M regulated glucose metabolic reprogramming by constitutively activating NF-κB to trigger inflammatory response, both of which were consistent with our results. Also, an earlier report suggested methylxanthines as a potential biomarker for NEC, indicating an accumulation of purine catabolic products in NEC patients [46]. Collectively, our results could reflect clinical scenarios characterized by devastating intestinal injury due to impaired nitrogen and purine nucleotide metabolism, and lethal inflammation due to aberrant glucose metabolism.

The intestinal barrier serves as the body’s first line of defense against foreign toxins. Tight and adherens junctions are crucial components of the mechanical barrier within the intestinal barrier, maintaining the integrity and permeability of the intestinal mucosal barrier [47]. These junctions are integral to the ECM. Tight junctions are located at the apical region of intercellular connections, while adherens junctions are situated basally [47]. Our results demonstrated that most extracellular proteins were upregulated, and thus suggested a reconstructed barrier during the short-term progression of NEC. Glycosylation, a posttranscriptional modification critical for cell attachment to the ECM [48], was significantly elevated, as evidenced by the substantial increase in UGGT1 level. A significant increase in CRHBP level suggested its role in ECM development during gestation [21]. Dipeptidases, which might cleave leukotriene D4 and thereby modulate inflammatory response and ECM activities [22, 49], showed an increase level of DPEP2 in our findings. MMP9, a known ECM-degrading enzyme, mediates inflammatory response and contributes to fetal membrane damage [50]. We observed an increased in MMP9 level during NEC progression, and its involvement in cancer [51], endocrine disorder [52], infection [53], and relaxin signaling [54] was noted. Interestingly, we observed a reduction in CD11A, which could accelerate neutrophil accumulation [55]. Taken together, these results might reflect clinical themes characterized by complex would healing and inflammation processes due to mixed ECM activities and immune responses in the short-term progression of NEC.

GPX3 functions as an antioxidant enzyme in inflammatory diseases and cancers [56, 57]. In this study, we observed a reduction in GPX3 expression, which may contribute to dysregulated immune response during NEC progression. GAG is abundant in ECM, and its synthesis relies on chain polymerization of heparan sulfate mediated by EXT1 and EXT2 [58]. Of note, GAG within human milk is considered as a biomarker in suppressing NEC [59]. We identified a potential reduction in EXT2 level during NEC progression, which could attenuate GAG synthesis. GAG has been shown to accelerate intestinal development during homeostasis [60]. Nevertheless, GAG may exert both pro-inflammatory and anti-inflammatory effects during inflammation [61]. The observed decrease in GAG level in our study might also reflect this dual role. Moreover, NEC progression is associated with an adaptive immune response characterized by B-cell activity [62]; however, the role of B cells in NEC progression remains controversial. B-cell-mediated Fc-γ receptor (Fc-γR)-dependent phagocytosis or IgA production can defend against intestinal bacteria and maintain homeostasis [63]. However, the possible dual effects of B cells should be considered, as evidence shows that B cells can also induce tissue lesion [62, 64, 65]. In our context of NEC progression, we identified a dysregulated B-cell immune response, as indicated by mixed changes of B-cell biomarkers and immunoglobulins. Interestingly, we detected an increase in cysteine-type endopeptidase activity, suggesting a potential apoptotic process during NEC progression [66]. Additionally, the altered levels of CUBN and APOB might contribute to disrupted lipid metabolism, affecting vitamin digestion and absorption [67, 68]. Collectively, these findings might reflect clinical scenarios characterized by immune dysregulation and impaired would healing due to mixed B-cell immune activities and reduced GAG level during long-term progression of NEC.

Several limitations of this study should be noted. The number of NEC patients included was limited, which might impact the generalizability of the findings. While we used external datasets to validate our results, future studies should aim to expand the sample size to enhance statistical power and robustness. Furthermore, due to certain constraints within our hospital, we encountered difficulties in conducting a number of biological experiments to investigate mechanistic details. Our future research should not only increase the sample size but also incorporate more experimental investigations to strengthen and validate these findings. To illustrate, we plan to genetically knockdown or use antibodies to interfere some critical DEPs in bowel epithelial cells for functional study to further enhance the biological credibility of our study.

In summary, our proteomic DIA analysis identified DEPs in NEC infants at various time points, revealing reduced protein, heme, nitrogen, and purine nucleotide biosynthesis during NEC formation. The study also suggested reconstructed ECM, aberrant B-cell immune responses, and reduced GAG level during NEC progression. These results were carefully validated and contextualized using external datasets and previous studies. Our work underscores the potential of DIA technology in evaluating and predicting NEC.

## Conclusion

Our comprehensive proteomic analysis not only demonstrates key pathways driving NEC but also establishes DIA MS as a powerful, noninvasive tool for assessing NEC formation and progression. This technology shows potential for broader clinical application, particularly in improving early diagnosis and identifying potential therapeutic targets for NEC. Future studies could further explore its utility in routine clinical practice and investigate its long-term impact on NEC management. These findings pave the way for future advancements in both diagnostic precision and personalized interventions, ultimately improving patient outcomes in NEC treatment.

## Supplementary Information

Below is the link to the electronic supplementary material.

Supplementary file1 (DOCX 1793 KB)

## Data Availability

The original contributions in this study are available from the corresponding author on reasonable request. The external datasets including (Mackay S. et al. [18]), GSE46619, and GSE62208 presented in this study can be found in online repositories.

## Abbreviations

AGC: Automatic gain control
BP: Biological process
CC: Cell component
COG: Cluster of orthologous groups
DEPs: Differentially expressed proteins
DIA: Data-independent acquisition
DTT: Dithiothreitol
ECM: Extracellular matrix
EDTA: Ethylenediaminetetraacetic acid
FA: Formic acid
FC: Fold change
FXR: Farnesoid X–activated receptor
GAG: Glycosaminoglycan
GO: Gene Ontology
HCD: Higher-energy collisional dissociation
HPLC: High-performance liquid chromatography
KEGG: Kyoto Encyclopedia of Genes and Genomes
MF: Molecular function
MS: Mass spectrometry
NEC: Necrotizing enterocolitis
NICU: Neonatal intensive care unit
PSMs: Peptide spectrum matches
QC: Quality control
SDS‒PAGE: Sodium dodecyl sulfate–polyacrylamide gel electrophoresis

## Declarations

### Source of biological material

The biological material used in this study was obtained from blood samples of preterm infants diagnosed with NEC. All samples were collected in accordance with ethical guidelines and with the informed consent of the parents or legal guardians. The study protocol was approved by the Ethics Committee of Shanghai Children’s Hospital, ensuring compliance with the ethical standards of research involving human subjects.

## References

[1] Masi AC, Embleton ND, Lamb CA, Young G, Granger CL, Najera J, et al. Human milk oligosaccharide DSLNT and gut microbiome in preterm infants predicts necrotising enterocolitis. Gut. 2021;70(12):2273–82. 10.1136/gutjnl-2020-322771. 33328245 10.1136/gutjnl-2020-322771 PMC9231288

[2] Miller J, Tonkin E, Damarell RA, McPhee AJ, Suganuma M, Suganuma H, et al. A systematic review and meta-analysis of human milk feeding and morbidity in very low birth weight infants. Nutrients. 2018;10(6):707. 10.3390/Nu10060707. 29857555 10.3390/nu10060707 PMC6024377

[3] Bethell GS, Knight M, Hall NJ. BAPS-CASS B-CNIGobo. Surgical necrotizing enterocolitis: association between surgical indication, timing, and outcomes. J Pediatr Surge. 2021;56(10):1785–90. 10.1016/j.jpedsurg.2021.04.028. 10.1016/j.jpedsurg.2021.04.028 34090670

[4] Elfvin A, Dinsdale E, Wales PW, Moore AM. Low birthweight, gestational age, need for surgical intervention and gram-negative bacteraemia predict intestinal failure following necrotising enterocolitis. Acta Paediatr. 2015;104(8):771–6. 10.1111/apa.12997. 25762289 10.1111/apa.12997

[5] Karila K, Anttila A, Iber T, Pakarinen M, Koivusalo A. Outcomes of surgery for necrotizing enterocolitis and spontaneous intestinal perforation in Finland during 1986–2014. J Pediatr Surge. 2018;53(10):1928–32. 10.1016/j.jpedsurg.2018.07.020. 10.1016/j.jpedsurg.2018.07.020 30122449

[6] Song J, Li Z, Yao G, Wei S, Li L, Wu H. Framework for feature selection of predicting the diagnosis and prognosis of necrotizing enterocolitis. PLoS One. 2022;17(8): e0273383. 10.1371/journal.pone.0273383. 35984833 10.1371/journal.pone.0273383 PMC9390903

[7] Nino DF, Sodhi CP, Hackam DJ. Necrotizing enterocolitis: new insights into pathogenesis and mechanisms. Nat Rev Gastro Hepat. 2016;13(10):590–600. 10.1038/nrgastro.2016.119. 10.1038/nrgastro.2016.119 PMC5124124 27534694

[8] Gagne D, Shajari E, Thibault MP, Noel JF, Boisvert FM, Babakissa C, et al. Proteomics profiling of stool samples from preterm neonates with SWATH/DIA mass spectrometry for predicting necrotizing enterocolitis. Int J Mol Sci. 2022;23(19):11601. 10.3390/ijms231911601. 36232903 10.3390/ijms231911601 PMC9569884

[9] Ng PC, Ang IL, Chiu RW, Li K, Lam HS, Wong RP, et al. Host-response biomarkers for diagnosis of late-onset septicemia and necrotizing enterocolitis in preterm infants. J Clin Invest. 2010;120(8):2989–3000. 10.1172/JCI40196. 20592468 10.1172/JCI40196 PMC2912182

[10] Zhao X, Zhou J, Liang W, Sheng Q, Lu L, Chen T, et al. Probiotics mixture reinforces barrier function to ameliorate necrotizing enterocolitis by regulating PXR-JNK pathway. Cell Biosci. 2021;11(1):20. 10.1186/s13578-021-00530-7. 33482929 10.1186/s13578-021-00530-7 PMC7824920

[11] Li P, Dong X, Wang XY, Du T, Du XJ, Wang S. Comparative proteomic analysis of adhesion/invasion related proteins in *Cronobacter sakazakii* based on data-independent acquisition coupled with LC-MS/MS. Front Microbiol. 2020;11:1239. 10.3389/fmicb.2020.01239. 32582128 10.3389/fmicb.2020.01239 PMC7296052

[12] Chatziioannou AC, Wolters JC, Sarafidis K, Thomaidou A, Agakidis C, Govorukhina N, et al. Targeted LC-MS/MS for the evaluation of proteomics biomarkers in the blood of neonates with necrotizing enterocolitis and late-onset sepsis. Anal Bioanal Chem. 2018;410(27):7163–75. 10.1007/s00216-018-1320-3. 30141021 10.1007/s00216-018-1320-3

[13] Sylvester KG, Ling XB, Liu GY, Kastenberg ZJ, Ji J, Hu Z, et al. Urine protein biomarkers for the diagnosis and prognosis of necrotizing enterocolitis in infants. The Journal of Pediatrics. 2014;164(3):607-12 e1-7. 10.1016/j.jpeds.2013.10.091. 24433829 10.1016/j.jpeds.2013.10.091 PMC4161235

[14] Pan X, Muk T, Ren S, Nguyen DN, Shen RL, Gao F, et al. Blood transcriptomic markers of necrotizing enterocolitis in preterm pigs. Pediatr Res. 2022;91(5):1113–20. 10.1038/s41390-021-01605-4. 34112973 10.1038/s41390-021-01605-4

[15] Wilhelm M, Schlegl J, Hahne H, Gholami AM, Lieberenz M, Savitski MM, et al. Mass-spectrometry-based draft of the human proteome. Nature. 2014;509(7502):582–7. 10.1038/nature13319. 24870543 10.1038/nature13319

[16] Lin PW, Stoll BJ. Necrotising enterocolitis. The Lancet. 2006;368(9543):1271–83. 10.1016/s0140-6736(06)69525-1. 10.1016/S0140-6736(06)69525-1 17027734

[17] Duchon J, Barbian ME, Denning PW. Necrotizing enterocolitis. Clin Perinatol. 2021;48(2):229–50. 10.1016/j.clp.2021.03.002. 34030811 10.1016/j.clp.2021.03.002

[18] Mackay S, Frazer LC, Bailey GK, Miller CM, Gong Q, Dewitt ON, et al. Identification of serum biomarkers for necrotizing enterocolitis using aptamer-based proteomics. Front Pediatr. 2023;11:1184940. 10.3389/fped.2023.1184940. 37325361 10.3389/fped.2023.1184940 PMC10264655

[19] Gao T, Hu S, Xu W, Wang Z, Guo T, Chen F, et al. Targeted LC-MS/MS profiling of bile acids reveals primary/secondary bile acid ratio as a novel biomarker for necrotizing enterocolitis. Anal Bioanal Chem. 2024;416(1):287–97. 10.1007/s00216-023-05017-7. 37938412 10.1007/s00216-023-05017-7 PMC10758366

[20] Yue Z, Yu Y, Gao B, Wang D, Sun H, Feng Y, et al. Advances in protein glycosylation and its role in tissue repair and regeneration. Glycoconj J. 2023;40(3):355–73. 10.1007/s10719-023-10117-8. 37097318 10.1007/s10719-023-10117-8

[21] Parets SE, Conneely KN, Kilaru V, Fortunato SJ, Syed TA, Saade G, et al. Fetal DNA methylation associates with early spontaneous preterm birth and gestational age. PLoS One. 2013;8(6): e67489. 10.1371/journal.pone.0067489. 23826308 10.1371/journal.pone.0067489 PMC3694903

[22] Habib GM, Shi ZZ, Cuevas AA, Lieberman MW. Identification of two additional members of the membrane-bound dipeptidase family. FASEB J Off Public Feder Am Soc Exp Biol. 2003;17(10):1313–5. 10.1096/fj.02-0899fje. 10.1096/fj.02-0899fje 12738806

[23] Lin CH, Krisp C, Packer NH, Molloy MP. Development of a data independent acquisition mass spectrometry workflow to enable glycopeptide analysis without predefined glycan compositional knowledge. J Proteom. 2018;172:68–75. 10.1016/j.jprot.2017.10.011. 10.1016/j.jprot.2017.10.011 29069609

[24] Song S, Cong W, Zhou S, Shi Y, Dai W, Zhang H, et al. Small GTPases: structure, biological function and its interaction with nanoparticles. Asian J Pharm Sci. 2019;14(1):30–9. 10.1016/j.ajps.2018.06.004. 32104436 10.1016/j.ajps.2018.06.004 PMC7032109

[25] Becker CF, Marsac Y, Hazarika P, Moser J, Goody RS, Niemeyer CM. Functional immobilization of the small GTPase Rab6A on DNA-gold nanoparticles by using a site-specifically attached poly(ethylene glycol) linker and thiol place-exchange reaction. Chembiochem. 2007;8(1):32–6. 10.1002/cbic.200600422. 17121405 10.1002/cbic.200600422

[26] Rai A, Singh AK, Bleimling N, Posern G, Vetter IR, Goody RS. Rep15 interacts with several Rab GTPases and has a distinct fold for a Rab effector. Nat Commun. 2022;13(1):4262. 10.1038/s41467-022-31831-1. 35871249 10.1038/s41467-022-31831-1 PMC9308819

[27] Zhang Y, Li G, Zhao Y. Advances in the development of Rho GTPase inhibitors. Bioorg Med Chem. 2023;90: 117337. 10.1016/j.bmc.2023.117337. 37253305 10.1016/j.bmc.2023.117337

[28] Cheng S, Zhang M, Li W, Wang Y, Liu Y, He Q. Proteomic analysis of porcine alveolar macrophages infected with porcine circovirus type 2. J Proteom. 2012;75(11):3258–69. 10.1016/j.jprot.2012.03.039. 10.1016/j.jprot.2012.03.039 22513220

[29] Bardell D, Milner PI, Goljanek-Whysall K, Peffers MJ. Differences in plasma and peritoneal fluid proteomes identifies potential biomarkers associated with survival following strangulating small intestinal disease. Equine Vet J. 2019;51(6):727–32. 10.1111/evj.13094. 30854696 10.1111/evj.13094

[30] Fagundes DJ, Carrara FL, Teixeira WA, Simoes RS, Taha MO. The role of the exogenous supply of adenosine triphosphate in the expression of Bax and Bcl2L1 genes in intestinal ischemia and reperfusion in rats 1. Acta Cir Bras. 2018;33(10):889–95. 10.1590/s0102-865020180100000003. 30484498 10.1590/s0102-865020180100000003

[31] Bunnell TM, Burbach BJ, Shimizu Y, Ervasti JM. Beta-actin specifically controls cell growth, migration, and the G-actin pool. Mol Biol Cell. 2011;22(21):4047–58. 10.1091/mbc.E11-06-0582. 21900491 10.1091/mbc.E11-06-0582 PMC3204067

[32] Reedy CJ, Gibney BR. Heme protein assemblies. Chem Rev. 2004;104(2):617–49. 10.1021/cr0206115. 14871137 10.1021/cr0206115

[33] Walter S, Mertens C, Muckenthaler MU, Ott C. Cardiac iron metabolism during aging - role of inflammation and proteolysis. Mech Ageing Dev. 2023;215: 111869. 10.1016/j.mad.2023.111869. 37678569 10.1016/j.mad.2023.111869

[34] Patel HJ, Patel BM. TNF-alpha and cancer cachexia: molecular insights and clinical implications. Life Sci. 2017;170:56–63. 10.1016/j.lfs.2016.11.033. 27919820 10.1016/j.lfs.2016.11.033

[35] Huang CM, Lowes MA, Cserti C, Alavi A. Hemoglobin levels and serum C-reactive protein in patients with moderate to severe hidradenitis suppurativa. J Cutan Med Surg. 2019;23(5):501–6. 10.1177/1203475419858963. 31253065 10.1177/1203475419858963

[36] Chokshi NK, Guner YS, Hunter CJ, Upperman JS, Grishin A, Ford HR. The role of nitric oxide in intestinal epithelial injury and restitution in neonatal necrotizing enterocolitis. Semin Perinatol. 2008;32(2):92–9. 10.1053/j.semperi.2008.01.002. 18346532 10.1053/j.semperi.2008.01.002 PMC2390779

[37] Kose FA, Pabuccuoglu A, Karakoyun M, Aydogdu S. Peroxiredoxins and hypoxia-inducible factor-1alpha in duodenal tissue: emerging factors in the pathophysiology of pediatric celiac disease patients. Curr Issues Mol Biol. 2023;45(2):1779–93. 10.3390/cimb45020114. 36826059 10.3390/cimb45020114 PMC9954839

[38] Rentea RM, Guo Y, Zhu X, Musch MW, Chang EB, Gourlay DM, et al. Role of intestinal Hsp70 in barrier maintenance contribution of milk to the induction of Hsp70.2. Pediatr Surge Int. 2018;34(3):323–30. 10.1007/s00383-017-4211-3. 10.1007/s00383-017-4211-3 29196880

[39] Gershner GH, Hunter CJ. Redox chemistry: implications for necrotizing enterocolitis. Int J Mol Sci. 2024;25(15):8416. 10.3390/ijms25158416. 39125983 10.3390/ijms25158416 PMC11312856

[40] Abu Faddan NH, Sherif TM, Mohammed OA, Nasif KA, El Gezawy EM. Intestinal barrier integrity and function in infants with cholestasis. Intest Res. 2017;15(1):118–23. 10.5217/ir.2017.15.1.118. 28239322 10.5217/ir.2017.15.1.118 PMC5323301

[41] Geleziunas R, McQuillan A, Malapetsa A, Hutchinson M, Kopriva D, Wainberg MA, et al. Increased DNA synthesis and repair-enzyme expression in lymphocytes from patients with chronic lymphocytic leukemia resistant to nitrogen mustards. J Nat Cancer Inst. 1991;83(8):557–64. 10.1093/jnci/83.8.557. 2005641 10.1093/jnci/83.8.557

[42] Jiang P, Sangild PT. Intestinal proteomics in pig models of necrotising enterocolitis, short bowel syndrome and intrauterine growth restriction. Proteom Clin Appl. 2014;8(9–10):700–14. 10.1002/prca.201300097. 10.1002/prca.201300097 24634357

[43] Saha SK, Goswami A, Dutta C. Association of purine asymmetry, strand-biased gene distribution and PolC within firmicutes and beyond: a new appraisal. BMC Genomics. 2014;15(1):430. 10.1186/1471-2164-15-430. 24899249 10.1186/1471-2164-15-430 PMC4070872

[44] Huang CY, Kuo WT, Huang CY, Lee TC, Chen CT, Peng WH, et al. Distinct cytoprotective roles of pyruvate and ATP by glucose metabolism on epithelial necroptosis and crypt proliferation in ischaemic gut. J Physiol. 2017;595(2):505–21. 10.1113/JP272208. 27121603 10.1113/JP272208 PMC5233659

[45] Kumar S, Donti TR, Agnihotri N, Mehta K. Transglutaminase 2 reprogramming of glucose metabolism in mammary epithelial cells via activation of inflammatory signaling pathways. Int J Cancer. 2014;134(12):2798–807. 10.1002/ijc.28623. 24477458 10.1002/ijc.28623

[46] Nowicki PT, Oh W. Methylxanthines and necrotizing enterocolitis revisited. J Pediatr Gastroenterol Nutr. 1989;9(2):137–8. 10.1097/00005176-198908000-00001. 2809934 10.1097/00005176-198908000-00001

[47] Camilleri M, Madsen K, Spiller R, Greenwood-Van Meerveld B, Verne GN. Intestinal barrier function in health and gastrointestinal disease. Neurogastroenterology and Motility: the official journal of the European Gastrointestinal Motility Society. 2012;24(6):503–12. 10.1111/j.1365-2982.2012.01921.x. 10.1111/j.1365-2982.2012.01921.x PMC5595063 22583600

[48] Zhang J, Jamaluddin M, Zhang Y, Widen SG, Sun H, Brasier AR, et al. Type II epithelial-mesenchymal transition upregulates protein N-glycosylation to maintain proteostasis and extracellular matrix production. J Proteome Res. 2019;18(9):3447–60. 10.1021/acs.jproteome.9b00342. 31424945 10.1021/acs.jproteome.9b00342 PMC7195216

[49] Mao W, Wang K, Wu Z, Xu B, Chen M. Current status of research on exosomes in general, and for the diagnosis and treatment of kidney cancer in particular. J Exp Clin Cancer Res CR. 2021;40(1):305. 10.1186/s13046-021-02114-2. 34583759 10.1186/s13046-021-02114-2 PMC8477471

[50] Helmo FR, Alves EAR, Moreira RAA, Severino VO, Rocha LP, Monteiro M, et al. Intrauterine infection, immune system and premature birth. J Maternal-Fetal Neonat Med off j Eur Assoc Perinat Med Federat Asia Ocean Perinat Soc Int Soc Perinat Obstet. 2018;31(9):1227–33. 10.1080/14767058.2017.1311318. 10.1080/14767058.2017.1311318 28423971

[51] Fan HX, Li HX, Chen D, Gao ZX, Zheng JH. Changes in the expression of MMP2, MMP9, and ColIV in stromal cells in oral squamous tongue cell carcinoma: relationships and prognostic implications. J Exp Clinic Cancer Res CR. 2012;31(1):90. 10.1186/1756-9966-31-90. 10.1186/1756-9966-31-90 PMC3490717 23107277

[52] Nishiyama K, Ono M, Tsuno T, Inoue R, Fukunaka A, Okuyama T, et al. Protective effects of imeglimin and metformin combination therapy on beta-cells in db/db male mice. Endocrinology. 2023;164(8):BQAD09. 10.1210/endocr/bqad095. 10.1210/endocr/bqad095 37314160

[53] Zhu L, Xin YJ, He M, Bian J, Cheng XL, Li R, et al. Downregulation of miR-337-3p in hypoxia/reoxygenation neuroblastoma cells increases KCTD11 expression. J Biochem Mol Toxicol. 2024;38(4):e23685. 10.1002/jbt.23685. 38495002 10.1002/jbt.23685

[54] Howatt DA, Dajee M, Xie X, Moorleghen J, Rateri DL, Balakrishnan A, et al. Relaxin and matrix metalloproteinase-9 in angiotensin II-induced abdominal aortic aneurysms. Circ J. 2017;81(6):888–90. 10.1253/circj.CJ-17-0229. 28420827 10.1253/circj.CJ-17-0229 PMC5964022

[55] Stadnyk AW, Dollard C, Issekutz TB, Issekutz AC. Neutrophil migration into indomethacin induced rat small intestinal injury is CD11a/CD18 and CD11b/CD18 co-dependent. Gut. 2002;50(5):629–35. 10.1136/gut.50.5.629. 11950807 10.1136/gut.50.5.629 PMC1773205

[56] Sun Q, Mehl S, Renko K, Seemann P, Gorlich CL, Hackler J, et al. Natural autoimmunity to selenoprotein P impairs selenium transport in Hashimoto’s thyroiditis. Int J Mol Sci. 2021;22(23):13088. 10.3390/ijms222313088. 34884891 10.3390/ijms222313088 PMC8658221

[57] Singh L, Atilano S, Chwa M, Singh MK, Ozgul M, Nesburn A, et al. Using human ‘personalized’ cybrids to identify drugs/agents that can regulate chronic lymphoblastic leukemia mitochondrial dysfunction. Int J Mol Sci. 2023;24(13):11025. 10.3390/ijms241311025. 37446202 10.3390/ijms241311025 PMC10341973

[58] Sugahara K, Kitagawa H. Recent advances in the study of the biosynthesis and functions of sulfated glycosaminoglycans. Curr Opin Struct Biol. 2000;10(5):518–27. 10.1016/s0959-440x(00)00125-1. 11042448 10.1016/s0959-440x(00)00125-1

[59] Monzon N, Kasahara EM, Gunasekaran A, Burge KY, Chaaban H. Impact of neonatal nutrition on necrotizing enterocolitis. Semin Pediatr Surge. 2023;32(3):151305. 10.1016/j.sempedsurg.2023.151305. 10.1016/j.sempedsurg.2023.151305 PMC10750299 37257267

[60] Burge K, Bergner E, Gunasekaran A, Eckert J, Chaaban H. The role of glycosaminoglycans in protection from neonatal necrotizing enterocolitis: a narrative review. Nutrients. 2020;12(2):546. 10.3390/nu12020546. 32093194 10.3390/nu12020546 PMC7071410

[61] Farrugia BL, Lord MS, Melrose J, Whitelock JM. The role of heparan sulfate in inflammation, and the development of biomimetics as anti-inflammatory strategies. J Histochem Cytochem off j Histochem Soc. 2018;66(4):321–36. 10.1369/0022155417740881. 10.1369/0022155417740881 PMC5958377 29290153

[62] Zhang L, Sun L, Wu M, Huang J. Identification of inflammatory genes, pathways, and immune cells in necrotizing enterocolitis of preterm infant by bioinformatics approaches. Biomed Res Int. 2021;2021:5568724. 10.1155/2021/5568724. 33880370 10.1155/2021/5568724 PMC8046524

[63] Culbreath C, Tanner SM, Yeramilli VA, Berryhill TF, Lorenz RG, Martin CA. Environmental-mediated intestinal homeostasis in neonatal mice. J Surgic Res. 2015;198(2):494–501. 10.1016/j.jss.2015.04.002. 10.1016/j.jss.2015.04.002 25940157

[64] Bednarek J, Traxinger B, Brigham D, Roach J, Orlicky D, Wang D, et al. Cytokine-producing b cells promote immune-mediated bile duct injury in murine biliary atresia. Hepatology. 2018;68(5):1890–904. 10.1002/hep.30051. 29679373 10.1002/hep.30051 PMC6195851

[65] Chen J, Crispin JC, Tedder TF, Dalle Lucca J, Tsokos GC. B cells contribute to ischemia/reperfusion-mediated tissue injury. J Autoimmun. 2009;32(3–4):195–200. 10.1016/j.jaut.2009.02.021. 19342197 10.1016/j.jaut.2009.02.021 PMC3734555

[66] Shi B, Lyu CJ, Le ZK, Ji HS, Xiao Y, Zhang YY, et al. NLRP3 activation in macrophages promotes acute intestinal injury in neonatal necrotizing enterocolitis. World J Pediatr. 2024;20(2):153–64. 10.1007/s12519-023-00727-5. 37389784 10.1007/s12519-023-00727-5 PMC10884143

[67] Peloso GM, Demissie S, Collins D, Mirel DB, Gabriel SB, Cupples LA, et al. Common genetic variation in multiple metabolic pathways influences susceptibility to low HDL-cholesterol and coronary heart disease. J Lipid Res. 2010;51(12):3524–32. 10.1194/jlr.P008268. 20855565 10.1194/jlr.P008268 PMC2975725

[68] Liu X, Zhang X, Li L, Wang J, Chen Y, Wu L. Bioinformatics analysis of potential key genes and pathways in neonatal necrotizing enterocolitis. BMC Pediatr. 2022;22(1):658. 10.1186/s12887-022-03721-4. 36371157 10.1186/s12887-022-03721-4 PMC9652887

