# Supplementary material for: Data-independent acquisition-based blood proteomics unveils predictive biomarkers for neonatal necrotizing enterocolitis
Source: Anal Bioanal Chem. 2024 Nov 20;417(1):199–218. doi: 10.1007/s00216-024-05637-7 (PMC11695561; doi:10.1007/s00216-024-05637-7)
Supplement: Supplementary file 1 — Supplementary file1 (DOCX 1793 KB) [file 216_2024_5637_MOESM1_ESM.docx]

**Supplemental materials**

**
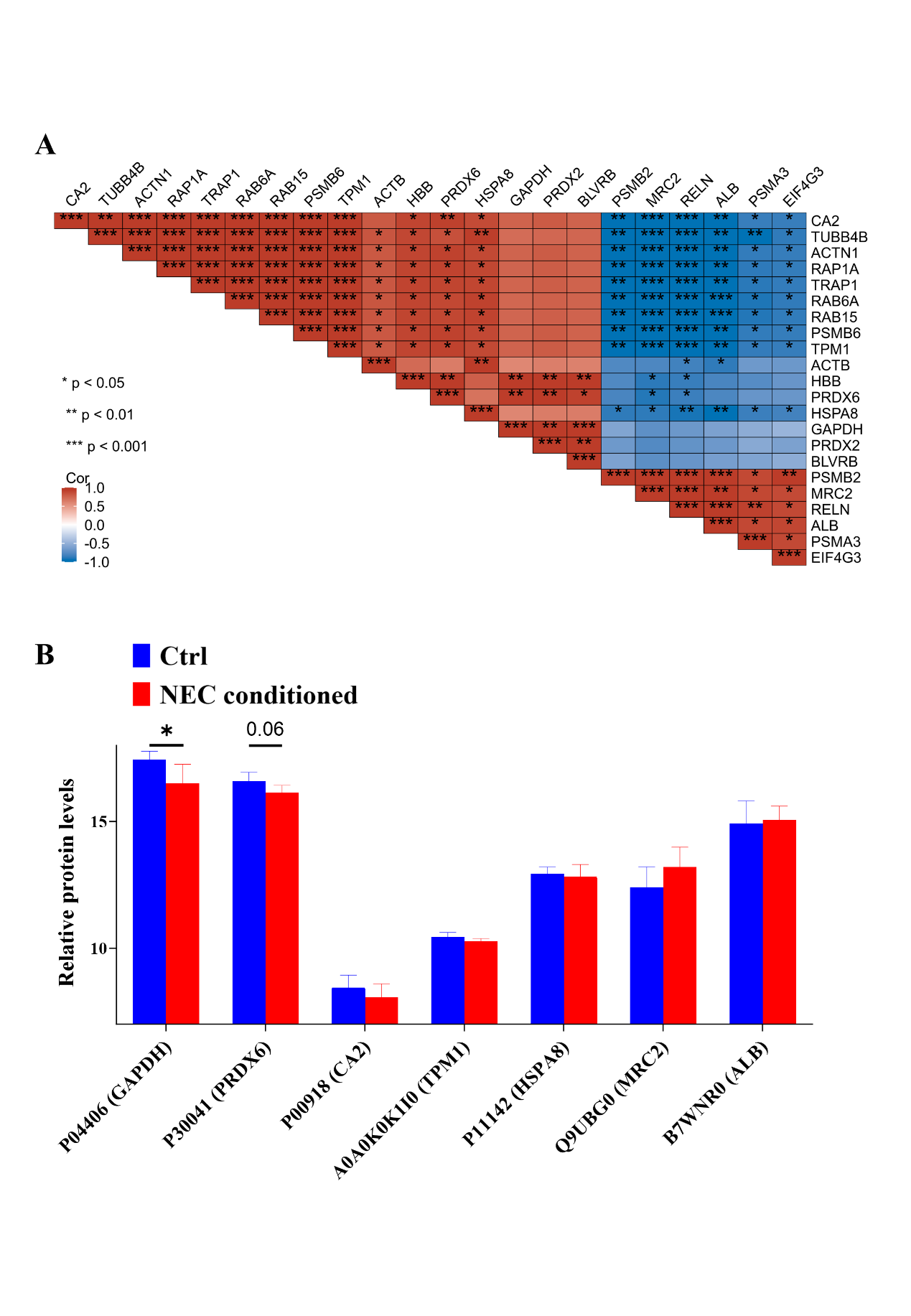
**

**Supplemental Figure 1. Correlation matrix with a proteomic external dataset providing validation of the DIA-MS findings to the early stage of NEC formation.**

***(A)*** *A matrix showing protein-protein correlations among hub DEPs during the early stage of NEC formation.* ***(B)*** *Box plots showing the altered proteins (Stephen et al. 2023) during the early stage of NEC formation.*

*Ctrl: control; DEP: differential expressed proteins; NEC: neonatal necrotizing enterocolitis. P<0.05 is shown as *, P<0.01 as ** and P<0.001 as ***.*

*
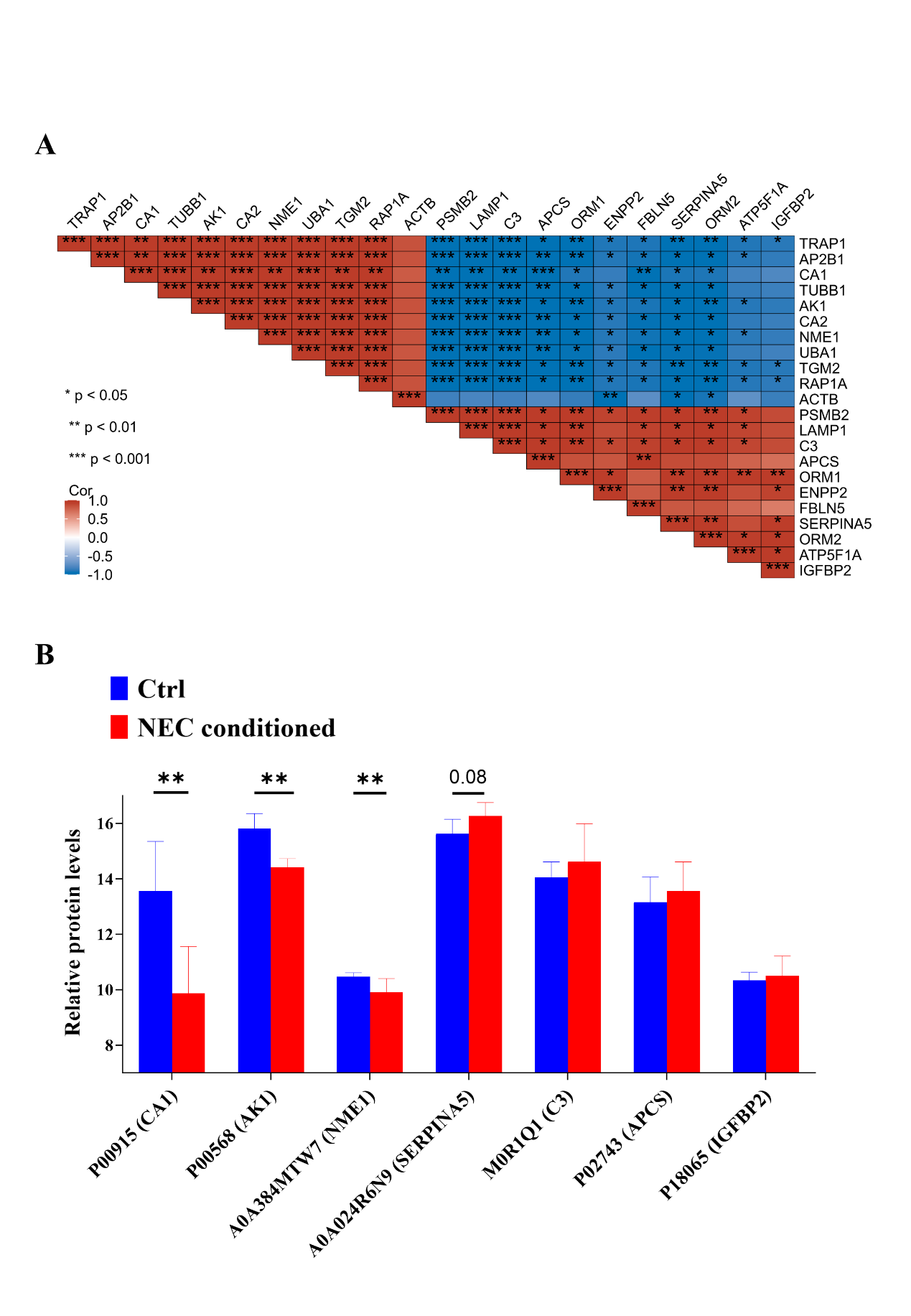
*

**Supplemental Figure 2. Correlation matrix with a proteomic external dataset providing validation of the DIA-MS findings to the late stage of NEC formation.**

***(A)*** *A matrix showing protein-protein correlations among hub DEPs during the late stage of NEC formation.* ***(B)*** *Box plots showing the altered proteins (Stephen et al. 2023) during the late stage of NEC formation.*

*Ctrl: control; DEP: differential expressed proteins; NEC: neonatal necrotizing enterocolitis. P<0.05 is shown as *, P<0.01 as ** and P<0.001 as ***.*

*
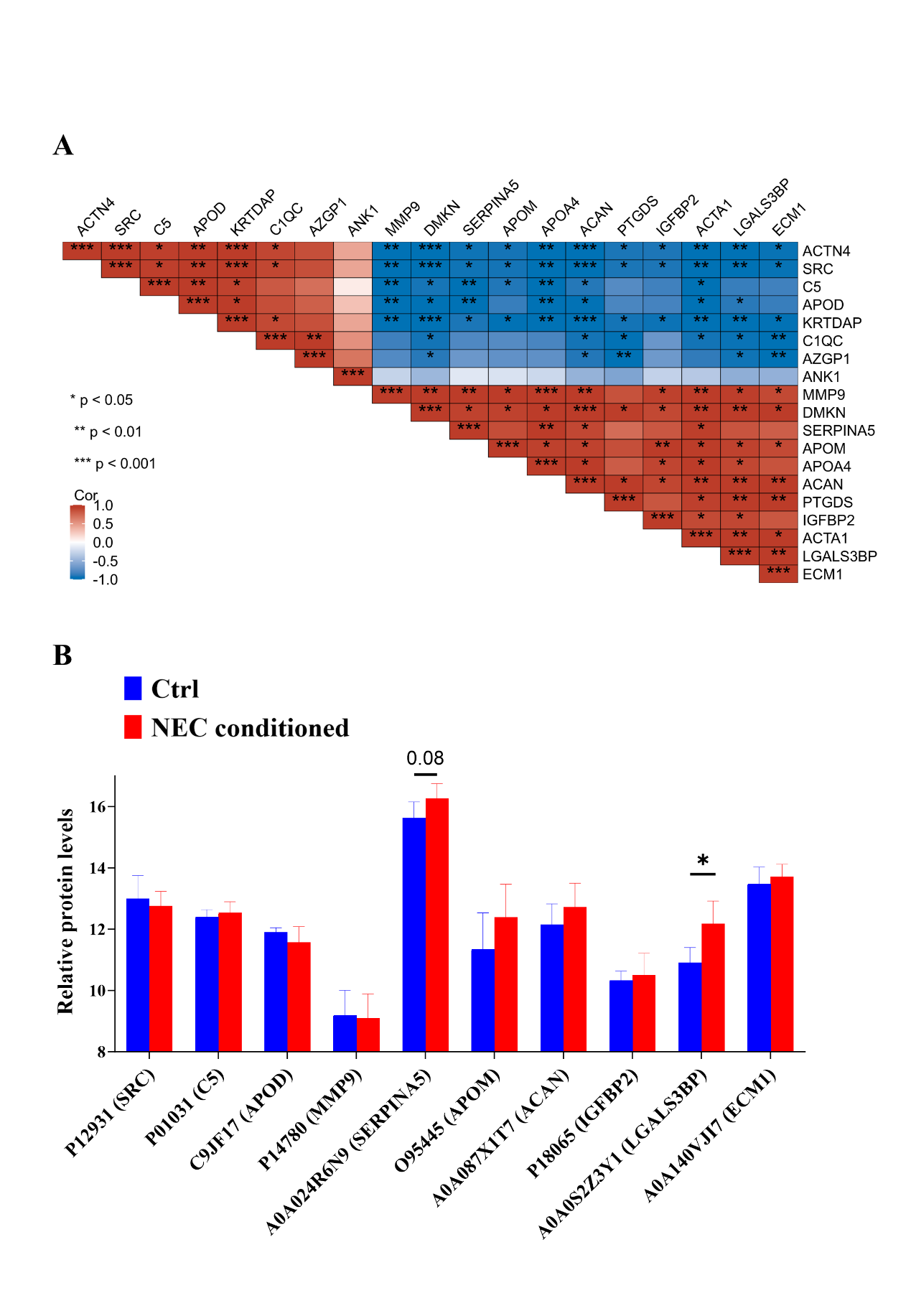
*

**Supplemental Figure 3. Correlation matrix with a proteomic external dataset providing validation of the DIA-MS findings in the short-term progression of NEC.**

***(A)*** *A matrix showing protein-protein correlations among hub DEPs during the late stage of NEC formation.* ***(B)*** *Box plots showing the altered proteins (Stephen et al. 2023) in the short-term progression of NEC.*

*Ctrl: control; DEP: differential expressed proteins; NEC: neonatal necrotizing enterocolitis. P<0.05 is shown as *, P<0.01 as ** and P<0.001 as ***.*

**
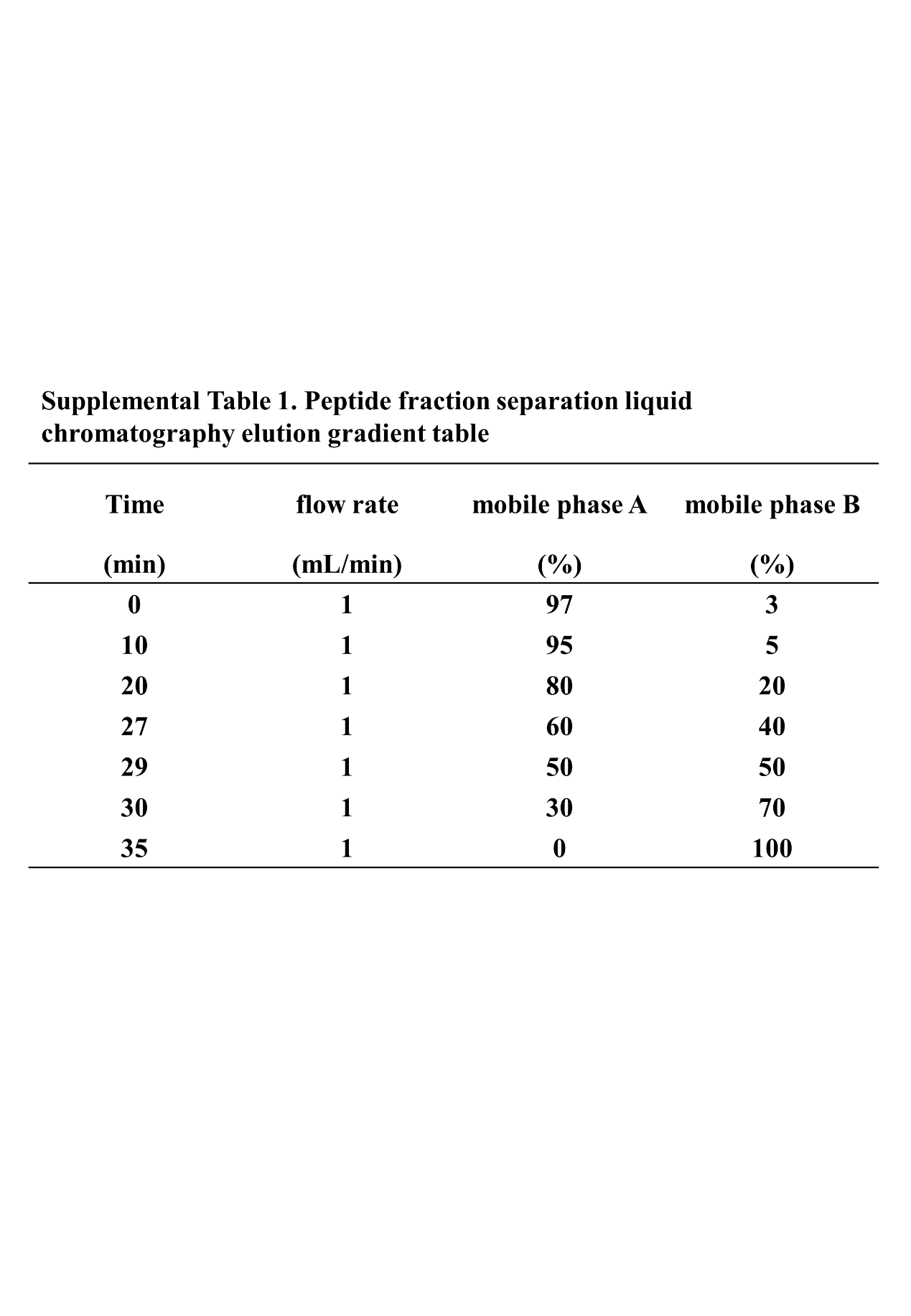
**

**Supplemental Table 1.** Peptide fraction separation liquid chromatography elution gradient table.


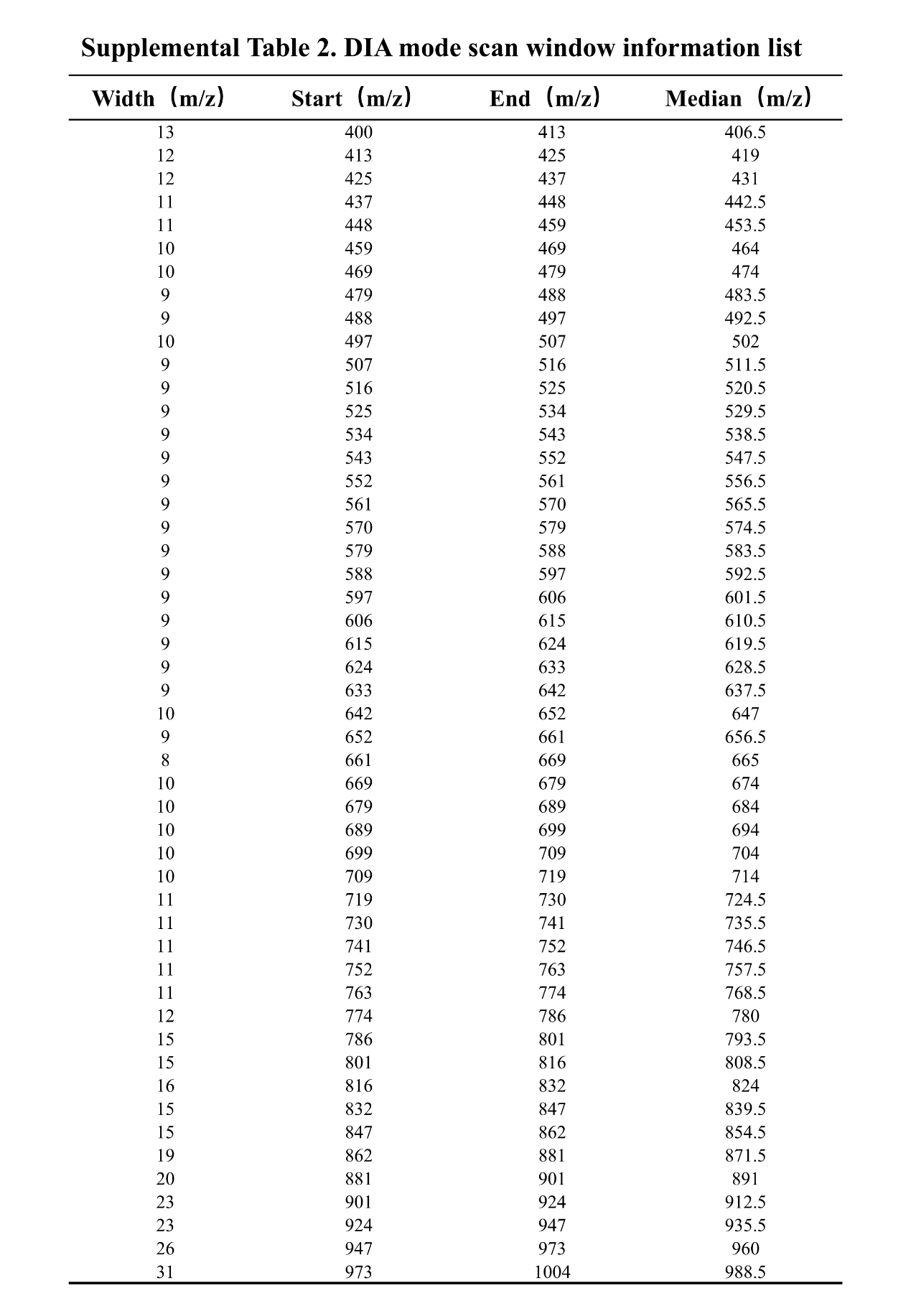
 **Supplemental Table 2.** DIA mode scan window information list.
